# M2-like tumor-associated macrophages transmit exosomal miR-27b-3p and maintain glioblastoma stem-like cell properties

**DOI:** 10.1038/s41420-022-01081-7

**Published:** 2022-08-04

**Authors:** Guifang Zhao, Lijuan Ding, Hongquan Yu, Weiyao Wang, Huan Wang, Yao Hu, Lingsha Qin, Guangce Deng, Buqing Xie, Guofeng Li, Ling Qi

**Affiliations:** 1 The Sixth Affiliated Hospital of Guangzhou Medical University, Qingyuan People’s Hospital, Qingyuan, 511518 People’s Republic of China; 2 Jilin Medical University, Jilin, 132013 People’s Republic of China; 3 Department of Radiation Oncology, the First Hospital of Jilin University, Changchun, 130021 People’s Republic of China; 4 Department of Oncological Neurosurgery, the First Hospital of Jilin University, Changchun, 130021 People’s Republic of China

**Keywords:** Cell biology, Diseases

## Abstract

There is growing evidence supporting the implications of exosomes-shuttled microRNAs (miRs) in the phenotypes of glioblastoma stem cells (GSCs), whilst the role of exosomal miR-27b-3p remains to be established. Herein, the aim of this study was to investigate the effect of M2 tumor-associated macrophage (TAM)-derived exosomal miR-27b-3p on the function of GSCs. Clinical glioblastoma (GBM) specimens were obtained and GSCs and M2-TAMs were isolated by fluorescence-activated cell sorting (FACS), and exosomes were separated from M2-TAMs. It was observed that M2-TAM-derived exosomes promoted the stem-like properties of GSCs. Gain- and loss- of function assays were then conducted to explore the effects of exosomal miR-27b-3p and the miR-27b-3p/MLL4/PRDM1 axis on GSC phenotypes. A xenograft tumor model of GBM was further established for in vivo substantiation. Inhibition of miR-27b-3p in M2-TAMs reduced exosomal miR-27b-3p transferred into GSCs and consequently diminished GSC viability in vitro and tumor-promoting effects of GSCs in vivo. The interaction among miR-27b-3p, mixed linked leukemia 4 (MLL4), positive regulatory domain I (PRDM1) was validated by dual-luciferase and ChIP assays. MLL4 positively regulated PRDM1 expression by inducing methylation in the PRDM1 enhancer region and ultimately reduced IL-33 expression. miR-27b-3p targeted MLL4/PRDM1 to activate IL-33 and maintain the stem-like function of GSCs. In conclusion, our study elucidated that M2-TAM-derived exosomal miR-27b-3p enhanced the tumorigenicity of GSCs through the MLL4/PRDM1/IL-33 axis.

## Introduction

Glioblastoma (GBM) is the most deadly subtype of primary brain tumors associated with a poor prognosis with a median survival shorter than 2 years [1]. Cancer stem cells (CSCs) are a sub-population of tumorigenic cells with high self-renewal potential at the apex of cellular hierarchies [2]. Notably, glioblastoma stem cells (GSCs) function as major contributors to the poor prognosis of GBM through supporting chemoresistance, radio-resistance, angiogenesis, and recurrence [3–5]. Tumor-associated macrophages (TAMs) produce factors that not only stimulate malignant behaviors of tumor cells but also enhance tumor vascularization [6]. Evidence exists revealing that TAMs may promote the invasiveness of GSCs [7]. Following previous documentation, this study aimed to further explore the function and mechanistic actions of TAMs and GSCs in GBM.

Extracellular vesicles (EVs) are membrane vesicles (including exosomes, microvesicles, and apoptotic bodies) capable to modulate the function of recipient cells by delivering RNAs, proteins and other molecular constituents [8]. For instance, enrichment of microRNA-21 (miR-21) in M2 bone marrow-derived macrophage-derived exosomes has been unraveled to promote the immune escape of glioma cells [9]. Intriguingly, it has been suggested that exosomes secreted by M2-TAMs contain high levels of miR-27b-3p [10], which has key roles to play in the pathogenesis of glioma [11]. miR-27b may raise the invasiveness of glioma cells by targeting Sprouty homolog 2 [12]. Herein, the modulatory effect of M2-TAMs may be associated with miR-27b-3p delivered by exosomes.

Furthermore, results of our bioinformatics analysis predicted mixed linked leukemia 4 (MLL4) as a target of miR-27b. In GBM, highly expressed histone H3 lysine 4 methyltransferase MLL4 prolongs the overall survival of patients [13]. Moreover, our co-expression analysis showed that the expression of MLL4 is positively correlated with that of positive regulatory domain I (PRDM1) in GBM. PRDM1 is a key component in the orderly transition from pluripotent state to defined neural lineages [14]. Interestingly, diminished PRDM1 expression is linked to poor survival and aggravated pathological grade of human glioma [15] and IL-33 is found to express heterogeneously in cancerous tissues and shares an association with inferior survival in patients suffered from relapse GBM [16]. More recently, a study indicated that IL-33 accelerates the invasion of glioma cells [17].

Based on the aforementioned evidence, this study intended to examine the impact of M2-TAM-derived exosomes-containing miR-27b-3p on GSCs and the underlying mechanisms likely associated with the MLL4/PRDM1/IL-33 regulatory axis.

## Results

### M2-TAMs promote the viability of GSCs

**Fig. 1:**
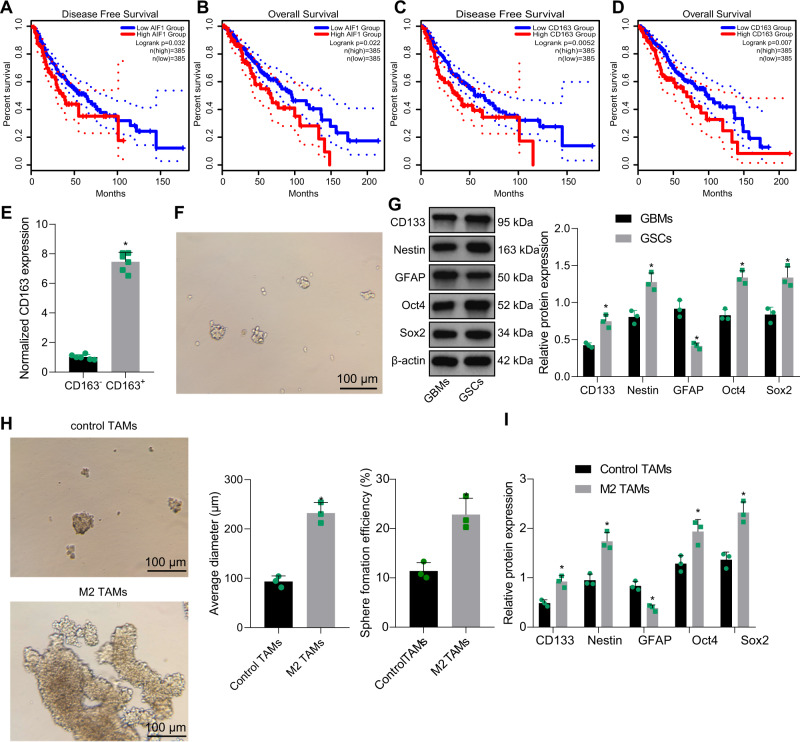
M2-TAMs maintain the properties of GSCs. Kaplan–Meier survival analysis of progression-free survival (**A**) and overall survival (**B**) of GBM patients with low or high Iba1 expression using GEPIA (http://gepia2.cancer-pku.cn/#index) in TCGA database (https://portal.gdc.cancer.gov/). Kaplan–Meier survival analysis of progression-free survival (**C**) and overall survival (**D**) of GBM patients with high or low CD163 expression using GEPIA in TCGA database. **E** RT-qPCR was utilized to analyze the expression of CD163 in the CD11b^+^/CD163^+^ and CD11b^+^/CD163^−^ TAMs from 6 GBM patients sorted by FACS. **F** The morphology of GSCs. **G** Western blot analysis of stem cell-related proteins CD133, Nestin, Oct4, Sox2, and GFAP. **H** Representative images showing neurosphere formation of GSCs along with the statistics of GSC neurosphere formation rate and diameter. **I** Expression of stem cell-related protein CD133, Nestin, Oct4, Sox2 and GFAP in GSCs measured by Western blot analysis. *p < 0.05 vs. CD163, GBMs or control TAMs. Measurement data were depicted as mean ± standard deviation, and comparison of data between two groups was conducted by unpaired *t* test. Cell experiments were repeated three times independently.

To further elucidate the molecular mechanism of M2-TAMs in tumor promotion, GEPIA was utilized to obtain the potential relationship between the survival rate of GBM patients and the expression of pan-TAM marker Iba1 (also named AIF1 in NCBI) and M2-TAM marker CD163 in TCGA database. Kaplan–Meier analysis results showed that GBM patients with lower expression of Iba1 or CD163 had a relatively better prognosis (Fig. 1A–D). Next, the tumor-supporting M2-TAMs (CD11b^+^/CD163^+^) and control TAMs (CD11b^+^/CD163^−^) were sorted by fluorescence-activated cell sorting (FACS) using the well-known TAM labeling CD11b and the classic M2-TAM labeling CD163. It was confirmed by reverse transcription quantitative polymerase chain reaction (RT-qPCR) that compared with control TAMs, the expression of CD163 was increased in M2-TAMs sorted by FACS (Fig. 1E). Accordingly, TAMs could be recruited by GSCs and maintained as M2-TAMs to promote GBM tumor growth.

Next, GSCs were isolated, the stemness of which was first assessed. Increasing evidence suggests that Oct4 and Sox2 are marker proteins of stem cells and tumor stem cells, which are essential for the maintenance of tumor stem cell stemness [5, 18, 19]. In addition, neural stem cell marker proteins CD133 and Nestin have also been found to be marker proteins of glioma stem cells, and their high expression is crucial for the self-renewal and proliferation of GSCs [18, 20]. GFAP is an intermediate filament protein, and its expression is related to the maturity of tumor cells. In the GSCs, GFAP signal is difficult to detect. However, GFAP is frequently highly expressed in mature glioma cells. Therefore, it is often used as a marker of GSC differentiation [21, 22]. GSCs were cultured in the nerve basal medium containing growth factor for suspension growth in a non-adherent culture system to form neurospheres (Fig. 1F). In contrast to the primary GBM cells, the levels of stem cell-related proteins CD133, Nestin, Oct4 and Sox2 were raised while that of astrocyte activation marker GFAP was diminished in GSCs (Fig. 1G).

In a Transwell system, GSCs were co-cultured with M2-TAMs or control TAMs, and the results reported that after co-culture with M2 TAMs, the formation rate and diameter of spheres in GSCs were increased obviously (Fig. 1H). The protein levels of CD133, Nestin, Oct4 and Sox2 were enhanced, while those of GFAP protein were reduced in GSCs co-cultured with M2-TAMs (Fig. 1I).

The above results suggested that M2-TAMs could enhance the viability of GSCs.

### M2-TAM-derived exosomes enhance the tumorigenic properties of GSCs

**Fig. 2:**
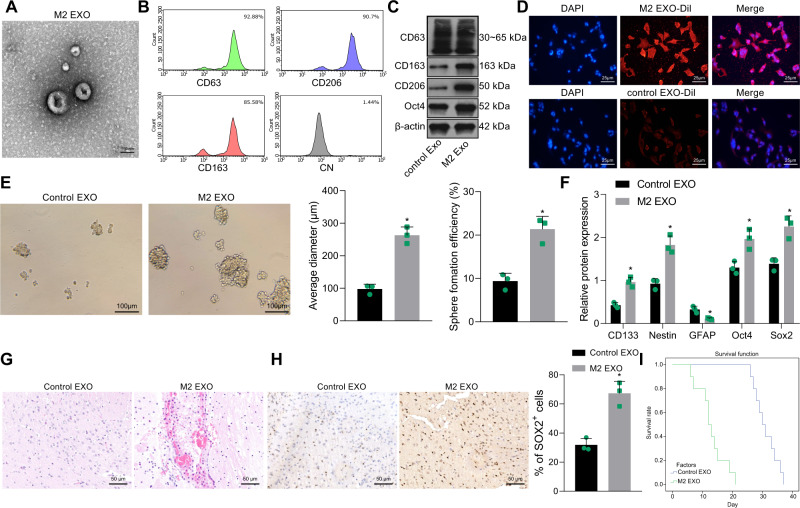
M2-TAM-derived exosomes enhance the stemness of GSCs. **A** TEM images of M2-TAM-derived exosomes. The presence of CD63, CD206, and CD163 in M2-TAM-derived exosomes analyzed by flow cytometric (**B**) and Western blot (**C**) analyses. **D** Fluorescence images of GSCs co-cultured with Dil-labeled M2-TAM-derived exosomes with nuclei stained with DAPI (blue). **E** Representative images showing neurosphere formation of GSCs along with the statistics of GSC formation rate and diameter of spheres. **F** Expression of stem cell-related protein CD133, Nestin, Oct4, Sox2, and GFAP in GSCs measured by Western blot analysis. **G** HE staining images of xenograft tumors from mice. **H** The representative immunohistochemical images of Sox2 in xenograft tumors and the percentage of GSCs labeled by Sox2. **I** Kaplan–Meier survive curve of tumor-bearing mice. *n* = 10. *p < 0.05 vs. the control EXO group. Measurement data were depicted as mean ± standard deviation, comparison of that between two groups was conducted by unpaired *t* test. Cell experiments were repeated three times independently.

M2-TAM-derived exosomes were isolated to investigate whether the exosomes secreted by M2-TAMs could promote the growth of GSCs as a paracrine pathway. The diameter of the M2-TAM-derived exosomes was about 180 nm as cup-shape under a transmission electron microscope (TEM) (Fig. 2A). Further flow cytometry and Western blot analyses suggested that M2 biomarkers (CD206 and CD163) and the exosome marker CD63 were enriched in M2-TAM-derived exosomes, while CD63 was enriched in the control TAM-derived exosomes (Fig. 2B, C).

To determine whether TAM-derived exosomes could be internalized by GSCs, M2-TAM-derived exosomes were labeled with CM-Dil and added to GSCs medium. As expected, the M2-TAM-derived exosomes were rapidly internalized into the cytoplasm of the GSCs (Fig. 2D). After co-culture with 40 μg/mL M2-TAM-derived exosomes, GSCs showed markedly increased formation rate and diameter of spheres (Fig. 2E). The protein levels of CD133, Nestin, Oct4 and Sox2 were heightened while GFAP level was diminished in GSCs co-cultured with M2-TAM-derived exosomes (Fig. 2F).

Next, the role of M2-TAM-derived exosomes was assessed in the mice bearing xenograft tumors of GSCs. M2-TAM-derived exosomes and GSCs were implanted into the brains of mice. Hematoxylin and eosin (HE) staining results revealed that versus the control TAM-derived exosomes, the tumorigenic role of GSCs was enhanced by M2-TAM-derived exosomes (Fig. 2G).

In order to investigate whether M2-TAM-derived exosomes affected the stemness maintenance of GSCs, the expression of GSCs marker Sox2 in xenograft was detected by immunohistochemical staining. In comparison to the control TAM-derived exosomes, the percentage of Sox2-labeled GSCs in xenograft was increased by M2-TAM-derived exosomes (Fig. 2H). Consequently, the survival time of mice treated with M2-TAM-derived exosomes was distinctly shortened (Fig. 2I).

Hence, M2-TAM-derived exosomes may be a key paracrine factor contributing to the tumorigenic properties of GSCs.

### miR-27b-3p transferred by M2-TAM-derived exosomes potentiates the tumorigenic properties of GSCs

**Fig. 3:**
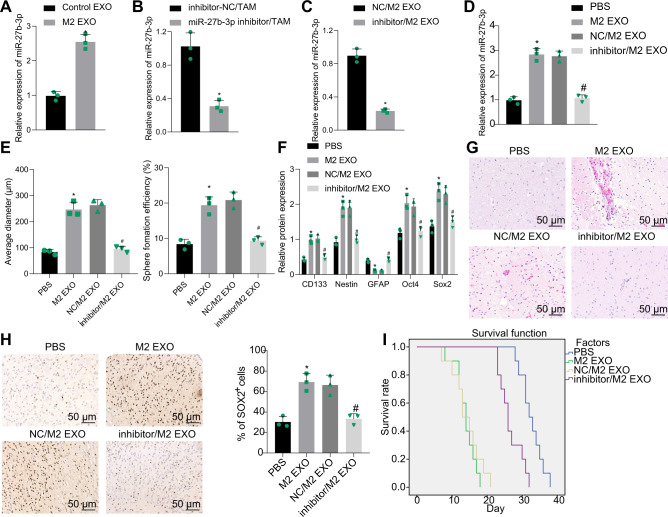
M2-TAM-derived exosomes containing miR-27b-3p enhance the properties of GSCs. **A** Expression of miR-27b-3p in M2-TAM-derived exosomes tested by RT-qPCR. **B** Expression of miR-27b-3p in M2-TAMs transfected with miR-27b-3p inhibitor tested by RT-qPCR. **C** Expression of miR-27b-3p in exosomes derived from M2-TAMs transfected with miR-27b-3p inhibitor analyzed by RT-qPCR. **D** Expression of miR-27b-3p in GSCs co-cultured with M2-TAM-derived exosomes. **E** The formation rate and diameter of spheres in GSCs. **F** Expression of stem cell-related protein CD133, Nestin, Oct4, Sox2, and GFAP in GSCs measured by Western blot analysis. **G** HE staining images of xenograft tumors from mice. **H** The representative immunohistochemical image of Sox2 in xenograft tumors and the percentage of GSC labeled by Sox2. **I** Kaplan–Meier survive curve of tumor-bearing mice. *n* = 10. *p < 0.05 vs. the control EXO group, the inhibitor-NC/TAM group, the NC/M2 EXO group, or the PBS group. # *P* < 0.05 vs. the NC/M2 EXO group. Measurement data were depicted as mean ± standard deviation. Comparison of data between two groups was conducted by unpaired *t* test, while that among multiple groups by one-way ANOVA followed by Tukey’s post hoc test. Cell experiments were repeated three times independently.

Exosomal miRNAs have been illustrated as important regulators of cellular functions [23]. Recently, miR-27b-3p was highly expressed in exosomes secreted by M2 macrophages [10]. Of note, miR-27b-3p expression was markedly increased in M2-TAM-derived exosomes relative to control TAM-derived exosomes (Fig. 3A).

It was verified that miR-27b-3p expression was diminished in the M2-TAMs transfected with miR-27b-3p inhibitor and their derived exosomes (Fig. 3B, C). Of note, the miR-27b-3p expression in the GSCs was enhanced by M2-TAM-derived exosomes, while miR-27b-3p expression in the GSCs was reduced by exosomes from the M2-TAMs that had been transfected with miR-27b-3p inhibitor (Fig. 3D).

Moreover, neurospheres formed in GSCs were increased by M2-TAM-derived exosomes, but decreased in response to miR-27b-3p inhibitor-treated M2-TAM-derived exosomes (Fig. 3E and Supplementary Fig. 1A). Besides, the protein levels of CD133, Nestin, Oct4, and Sox2 were raised, while GFAP level was reduced in GSCs after co-culture with M2-TAM-derived exosomes; however, miR-27b-3p inhibitor reversed the effects of M2-TAM-derived exosomes on the above proteins (Fig. 3F).

After the exosomes and GSCs were delivered into the brain of mice, the observation after HE staining showed that the tumor-promoting effect of GSCs was enhanced by M2-TAM-derived exosomes, but suppressed by the exosomes derived from the miR-27b-3p inhibitor-treated M2-TAMs (Fig. 3G). The immunohistochemical staining presented that the percentage of Sox2-labeled GSCs was increased by the M2-TAM-derived exosomes but it was reduced following miR-27b-3p inhibition (Fig. 3H). Additionally, the survival time of mice was shortened by treatment with M2-TAM-derived exosomes, but prolonged by the exosomes derived from the miR-27b-3p inhibitor-treated M2-TAMs (Fig. 3I).

Together, M2-TAM-derived exosomes can promote the properties of GSCs by delivering miR-27b-3p.

### Overexpression of MLL4 inhibits the properties of GSCs

**Fig. 4:**
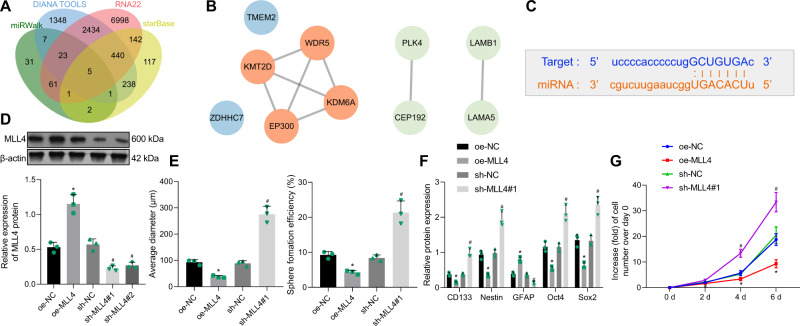
MLL4 inhibits the properties of GSCs. **A** Venn diagram of the target genes of miR-27b-3p predicted by the miRWalk (http://mirwalk.umm.uni-heidelberg.de/), DIANA TOOLS (http://diana.imis.athena-innovation.gr/DianaTools/index.php?r=microT_CDS/index), RNA22 (https://cm.jefferson.edu/rna22/) and starBase (http://starbase.sysu.edu.cn/) databases. **B** PPI network of the target genes and the related genes constructed using STRING database (https://string-db.org/). The core degree was calculated via Cytoscape (https://cytoscape.org/). Higher degree of gene core corresponds to redder color of the circle; conversely, lower degree of gene core corresponds to bluer circle. **C** The miR-27b-3p binding sites in the MLL4 3′UTR predicted by the starBase database. **D** The expression of MLL4 in GSCs in GSCs transfected with oe-MLL4 or sh-MLL4 measured by Western blot analysis. **E** The formation rate and diameter of spheres in GSCs transfected with oe-MLL4 or sh-MLL4. **F** Expression of stem cell-related proteins CD133, Nestin, Oct4, Sox2, and GFAP in GSCs transfected with oe-MLL4 or sh-MLL4 measured by Western blot analysis. **G** Viability of GSCs transfected with oe-MLL4 or sh-MLL4. *p < 0.05 vs. the oe-NC group. ^#^*p* < 0.05 vs. the sh-NC group. Measurement data were depicted as mean ± standard deviation. Comparison of data among multiple groups was conducted by one-way ANOVA followed by Tukey’s post hoc test. The data among multiple groups at different time points were compared using repeated-measures ANOVA with Bonferroni’s post hoc test. Cell experiments were repeated three times independently.

We next elucidated the molecular mechanism of miR-27b-3p in the maintenance of GSCs. miRWalk, DIANA TOOLS, RNA22, and starBase were utilized to predict the targeted genes of miR-27b-3p, and 131, 4496, 10,104, and 946 genes were obtained, respectively. Interestingly, 5 important downstream mRNAs were found in the intersection (Fig. 4A). STRING database was adopted to predict genes related to the aforementioned downstream mRNAs and to construct a protein-protein interaction (PPI) network. MLL4 (also named KMT2D in NCBI) had the highest core degree among the genes related to the downstream mRNAs (Fig. 4B), which was selected as key downstream gene for subsequent analyses. The binding site of miR-27b-3p and MLL4 was predicted by starBase (Fig. 4C).

MLL4 was overexpressed by oe-MLL4 transfection and knocked down by short hairpin RNA (sh)-MLL4 (sh-MLL4#1, sh-MLL4#2) transfection in GSCs to examine its role in GSCs. It was noted that sh-MLL4#1 which presented with the optimal silencing efficiency was selected for subsequent experiments (Fig. 4D). Consequently, neurosphere formation in GSCs was suppressed following overexpression of MLL4, while it was promoted following silencing of MLL4 (Fig. 4E and Supplementary Fig. 1B).

In addition, the protein levels of CD133, Nestin, Oct4, and Sox2 proteins were reduced while GFAP protein level was increased by MLL4 overexpression in GSCs, which were opposite to the changes caused by MLL4 knockdown (Fig. 4F). Meanwhile, the viability of GSCs was suppressed by MLL4 overexpression, but it was facilitated by MLL4 silencing (Fig. 4G).

Overall, overexpression of MLL4 attenuated the properties of GSCs, while its knockdown abolished this effect.

### miR-27b-3p targets MLL4 and negatively regulates its expression in GSCs

**Fig. 5:**
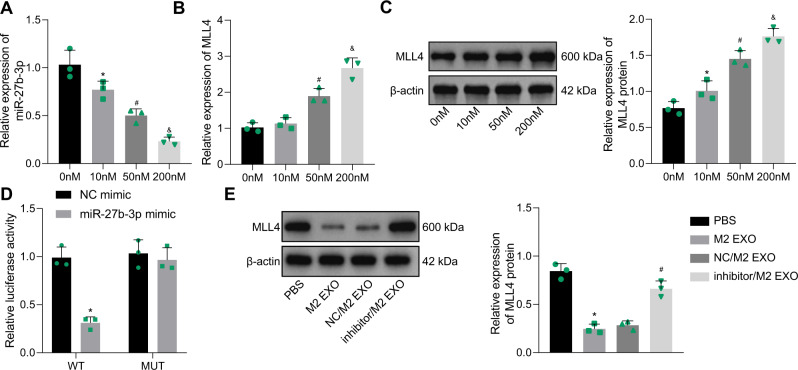
miR-27b-3p targets MLL4 and downregulates its expression. **A** After GSCs were treated with miR-27b-3p inhibitor (0, 10, 50, and 200 nM), the expression of miR-27b-3p in GSCs was determined by RT-qPCR. **B** After GSCs were treated with miR-27b-3p inhibitor (0, 10, 50, and 200 nM), the expression of MLL4 in GSCs was tested by RT-qPCR. **C** After GSCs were treated with miR-27b-3p inhibitor (0, 10, 50, and 200 nM), the protein expression of MLL4 in GSCs was measured by Western blot analysis. **D** The binding of miR-27b-3p to MLL4 tested by dual luciferase reporter assay. **E** After co-culture of GSCs with M2-TAM-derived exosomes or exosomes derived from miR-27b-3p inhibitor-treated M2-TAMs, the expression of MLL4 in GSCs as measured by western bot analysis. *p < 0.05 vs. the 0 nM group, the NC mimic group, or the PBS group. ^#^*p* < 0.05 vs. the 10 nM group or the M2 EXO group. ^&^*p* < 0.05 vs. the 50 nM group. Measurement data were depicted as mean ± standard deviation. Comparison of data between two groups was conducted by unpaired *t* test, while that among multiple groups by one-way ANOVA followed by Tukey’s post hoc test. Cell experiments were repeated three times independently.

In order to further determine the regulatory effect of miR-27b-3p on MLL4, GSCs were treated with a gradient concentration of miR-27b-3p inhibitor (0, 10, 50, and 200 nM). We demonstrated that the expression of miR-27b-3p was decreased in GSCs transfected with miR-27b-3p inhibitor in a dose-dependent manner (Fig. 5A).

In addition, MLL4 expression was detected to be increased at the mRNA and protein levels when miR-27b-3p expression was inhibited in a dose-dependent manner (Fig. 5B, C). More importantly, the luciferase activity of MLL4-WT was diminished in HEK-293 cells transfected with miR-27b-3p mimic; on the contrary, MLL4-MUT had no changes after transfection (Fig. 5D). These results suggested that miR-27b-3p could directly target MLL4 3′UTR.

GSCs were co-cultured with M2-TAM-derived exosomes and then with exosomes from miR-27b-3p inhibitor-treated M2-TAMs to examine whether M2-TAM-derived exosomes affected the expression of MLL4 by miR-27b-3p. Western blot analysis results indicated that M2-TAM-derived exosomes reduced the expression of MLL4, whereas miR-27b-3p inhibitor led to increased expression of MLL4 (Fig. 5E).

In summary, miR-27b-3p can bind to MLL4 and reduce its expression.

### miR-27b-3p enhances the properties of GSCs by downregulating the MLL4/PRDM1/IL-33 axis

**Fig. 6:**
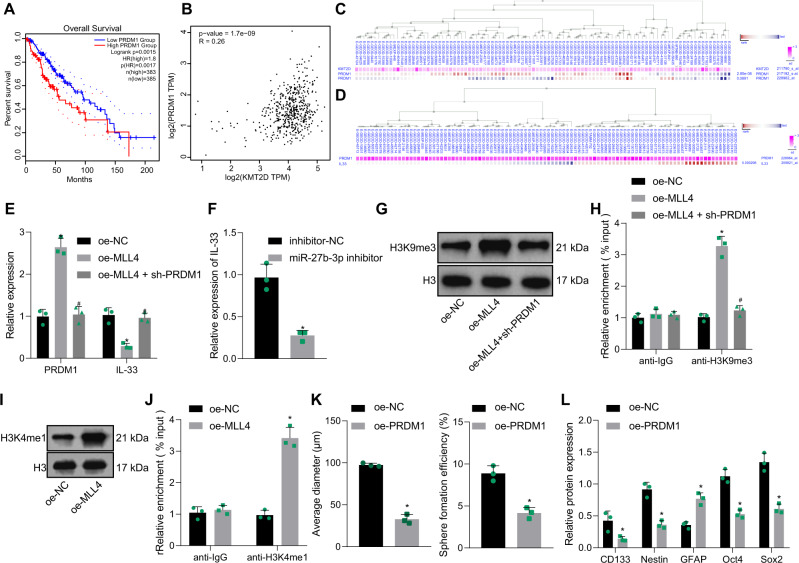
miR-27b-3p increases the properties of GSCs by downregulating the MLL4/PRDM1/IL-33 axis. **A** Survival curve of GBM patients with high or low PRDM1 expression. **B** Correlation between the expression of MLL4 and PRDM1 in GBM analyzed by GEPIA. Co-expression of MLL4 and PRDM1 (**C**) as well as PRDM1 and IL-33 (**D**) analyzed by the MEM database (https://biit.cs.ut.ee/mem/index.cgi). PRDM1 (**E**) and IL-33 (**E**, **F**) expression in GSCs determined by RT-qPCR. H3K4me1 (**I**) and H3K9me3 (**G**) levels in GSCs measured by Western blot analysis. The relative levels of IL-33 (**H**) and PRDM1 (**J**) analyzed by ChIP assay. **K** The formation rate and diameter of spheres in GSCs. **L** Expression of stem cell-related protein CD133, Nestin, Oct4, Sox2, and GFAP in GSCs measured by Western blot analysis. *p < 0.05 vs. the oe-NC group or the inhibitor-NC group. ^#^*p* < 0.05 vs. the oe-MLL4 group. Measurement data were depicted as mean ± standard deviation. Comparison of data between two groups was conducted by unpaired *t* test, while that among multiple groups by one-way ANOVA followed by Tukey’s post hoc test. Cell experiments were repeated three times independently.

PRDM1 has been reported to affect the progression of glioma [15], and IL-33 has been demonstrated to promote the stemness of tumor stem cells [24]. GEPIA analysis revealed a close correlation of PRDM1 expression with the survival of GBM patients (Fig. 6A), and a positive correlation was found between MLL4 expression and PRDM1 expression in GBM (Fig. 6B). MEM analysis presented a significant co-expression relationship between MLL4 expression and PRDM1 expression as well as PRDM1 expression and IL-33 expression (Fig. 6C, D).

GSCs were transfected with oe-MLL4 and the expression of IL-33 was determined to be reduced (Fig. 6E). Besides, miR-27b-3p inhibitor diminished the expression of IL-33 in GSCs (Fig. 6F). It was found that PRDM1 could recruit G9a to the IL-33 promoter, promoting H3K9 modification and inhibiting its transcription [25]. Thus, we speculated that MLL4 might downregulate IL-33 through PRDM1 and thus affected the functions of GSCs.

It was revealed that PRDM1 expression and H3K9me3 level were elevated in GSCs transfected with oe-MLL4 (Fig. 6E, G). Next, GSCs were transfected with oe-MLL4 or co-transfected with oe-MLL4 and sh-PRDM1. Consequently, PRDM1 expression and H3K9me3 level were diminished and IL-33 expression was elevated in GSCs co-transfected with oe-MLL4 and sh-PRDM1 as compared to oe-MLL4 treatment alone (Fig. 6E, G).

Finally, chromatin immunoprecipitation (ChIP) assay demonstrated that H3K9me3 recruited more IL-33 in the GSCs with oe-MLL4, while H3K9me3 recruited less IL-33 in the GSCs co-transfected with oe-MLL4 and sh-PRDM1 (Fig. 6H). The results of Western blot analysis that overexpression of MLL4 raised the level of H3K4me1 in GSCs (Fig. 6I). ChIP assay further demonstrated that upregulated MLL4 promoted the methylation of PRDM1 enhancer region (Fig. 6J). These results indicated that MLL4 downregulated IL-33 by increasing PRDM1.

The function of PRDM1 in GSCs was further verified using gain-of-function approaches. The formation rate and diameter of sphere of GSCs were attenuated by PRDM1 overexpression in GSCs (Fig. 6K and Supplementary Fig. 1C). Consistently, the levels of CD133, Nestin, Oct4, and Sox2 were diminished while GFAP expression was enhanced by PRDM1 overexpression in GSCs (Fig. 6L).

The above results suggested that miR-27b-3p could promote the properties of GSCs by downregulating the MLL4/PRDM1/IL-33 axis.

## Discussion

Our results provided new mechanistic insights for an understanding of the impact of M2-TAMs exosomal miR-27b-3p on the function of GSCs and the downstream mechanisms. M2-TAM-derived exosomes sustain the stem-like function of GSCs depending on miR-27b-3p. Besides, M2-TAM-derived exosomes increased the tumor-promoting effect of GSCs and shortened the survival time of mice with GBM by transmitting miR-27b-3p.

It was revealed by our study that M2-TAM-derived exosomes raised the formation rate and diameter of spheres in GSCs and potentiated their tumor-initiating effects. M2-TAMs have also been reported to correlate with oncogenesis and tumor progression. For instance, M2-TAMs are markedly related to the development of premalignant lesions to oral squamous cell carcinoma [26]. The EVs originating from M2-TAMs contain deaminase proteins or regulatory molecules of deaminase-specific transcription/translation which are implicated in cancer progression [27]. Another study suggested that tumor-derived exosomes from hypoxic conditions accelerate tumor growth and angiogenesis in GBM [28]. In this study, M2-TAM-derived exosomes facilitated tumorigenesis through maintenance of the GSCs, which was supported by the in vivo data from the murine model.

In addition, inhibition of miR-27b-3p in the M2-TAM-derived exosomes attenuated the stem-like properties and tumor-promoting effect of GSCs and consequently prolonged the survival time of mice with GBM. It has been suggested previously that upregulation of miR-27b-3p accelerates the proliferation and apoptosis resistance in myeloma fibroblasts [29]. miR-27b-3p functions as an oncogenic miR in colorectal cancer [29]. In addition, the expression of miR-27b-3p is heightened in Dox-resistant anaplastic thyroid cancer cells and its ectopic expression enhances the Dox resistance [30].

In this study, we further found that miR-27b-3p directly targeted MLL4 and negatively regulated its expression in GSCs. It is well-known that miRNAs promote the degradation of target mRNAs to inhibit the expression of the mRNAs [31]. Moreover, our study demonstrated that upregulating MLL4 reduced self-renewal and tumor-promoting properties of GSCs and inhibited their viability. It is reported that restoration of MLL4 positively influences the outcome of GBM patients [13]. Our study revealed that MLL4 expression was positively related to PRDM1 expression, and that MLL4 could promote PRDM1 transcription through the increase of the H3K4me1 enrichment at the enhancer region of PRDM1. As an H3K4 methylation methyltransferase, MLL4 often catalyzes H3K4me1/2 modification and H3K4me1/2 is widely distributed in the upstream enhancer region of genes to provide gene transcription [32, 33]. Further, previous literature has reported that PRDM1 can recruit G9a to the IL-33 promoter, thus promoting H3K9 modification, and inhibiting its transcription [25]. Meanwhile, G9a is a methyltransferase which catalyzes methylation of H3K9 [34]. In this study, we further explored it in GBM cells and found that it did exist, and had an important regulatory effect on GBM growth.

The bioinformatics and biological approaches of Wang’s study have demonstrated that the tumor-suppressive PRDM1 was a direct target gene of miR-30a-5p, and aberrant deficiency of PRDM1 was attributable to miR-30a-5p overexpression in GBM which contribute to phenotype maintenance and pathogenesis of gliomas [15]. Consistent with our findings, another study has indicated that PRDM1 could recruit G9a to the IL-33 promoter, promote H3K9 modification and inhibit its transcription [25]. It has been suggested that overexpression of PRDM1 inhibits cell proliferation, cell cycle arrest and enhances apoptosis of tumor cells [35]. Also, it is presented that upregulating PRDM1 in human colon cancer organoids can suppress the growth and formation of colon tumor organoids in vitro [36]. Our data substantiated that miR-27b-3p targeted MLL4/PRDM1 to activate IL-33 and thereby contributed to the maintenance of the stem-like function of GSCs. Nevertheless, the interactions among miR-27b-3p, MLL4, PRDM1, and IL-33 need further exploration.

**Fig. 7:**
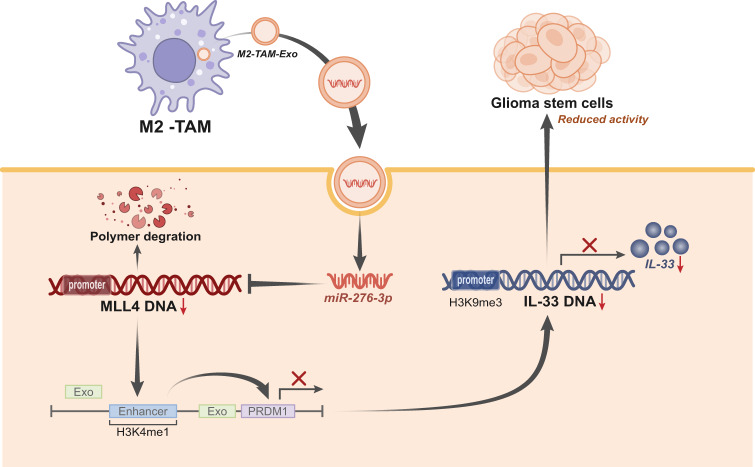
A molecular mechanism map illustrating the involvement of M2-TAM-derived exosomal miR-27b-3p in the properties of GSCs. M2-TAM-derived exosomal miR-27b-3p targets MLL4 and upregulates the PRDM1/IL-33 axis, thereby promoting the properties of GSCs.

In conclusion, our study provides evidence that M2-TAMs exosomal miR-27b-3p promotes the stem-like phenotype of GSCs *via* mediating the MLL4/PRDM1/IL-33 axis (Fig. 7). This study provides a new mechanistic insight for the development of GBM. However, a conclusion about the oncogenic effects of M2-TAMs exosomal miR-27b-3p may be limited by the lack of clinical data on this, which should be further probed in future studies.

## Materials and methods

### Ethics statement

The study protocols were approved by the Ethics Committee of Jilin Medical University. All the participants signed written informed consents. The experiments involving animals were in line with the Guide for the Care and Use of Laboratory Animal issued by the US National Institutes of Health.

### Study subjects

Tumor specimens were collected from 6 GBM patients who underwent resection at Jilin Medical University from 2017 to 2019. According to the World Health Organization (WHO) classification, the enrolled patients were histopathologically diagnosed with GBM. Fresh GBM specimens were collected for FACS to isolate GSCs and TAMs.

### Isolation of CD11b^+^/CD163^+^ M2-TAMs and CD11b^+^/CD163^−^ TAMs

The cells isolated from human GBM tumors were incubated with Alexa Fluor 488-conjugated mouse anti-human CD11b (R&D Systems, Minneapolis, MN; FAB16991G-100) and CD163-APC (Miltenyi Biotec GmbH, Bergisch Gladbach, Germany; 130-100-612) or control immunoglobulin G (IgG) (Miltenyi, 130-098-846 and R&D Systems, IC0041G) for 30 min at 4 °C. FACS was performed to sort TAMs using a BD FACSAria II cell sorter (Becton, Dickinson and Company, NJ).

### Isolation of GSCs

The fresh GBM tumor was separated by Papain separation system (Worthington Biochemical Corporation, Freehold, NJ). Cells were cultured for 6 h in nerve basal medium (Invitrogen, Carlsbad, CA) and B27 supplements (20 ng/mL, Thermo Fisher Scientific, Waltham, MA), epidermal growth factor (20 ng/mL, Peprotech, Rocky Hill, NJ) and basic fibroblast growth factor (20 ng/mL, Peprotech) to re-express GSC surface markers. Then, cells were labeled with fluorescein isothiocyanate-conjugated CD15 antibody (BD Biosciences, Franklin Lakes, NJ) and P-phycoerythrin (PE)-conjugated CD133 antibody (Miltenyi, 130-090-854) at 4 °C for 40 min. Next, GSCs (CD15^+^/CD133^+^) were isolated by FACS. The characteristics of GSC were verified using GSC markers Sox2, OLIG2, CD15, and CD133, and a series of functional tests, including tumor sphere formation, serum-induced differentiation, and in vivo limiting-dilution assays [37]. The enriched GSCs were continuously preserved as GBM xenograft, and the cells only cultured in vitro stem cell medium were used for functional experiments. The cells had been identified by karyotype and morphology. All the cells were tested by RT-qPCR for mycoplasma contamination and confirmed to be mycoplasma-free.

### Cell co-culture

Using a Transwell co-culture system (Corning), M2-TAMs were co-cultured with GSCs, while TAMs without co-culture were used as the control. Cells were co-cultured for 24 h and then adopted for neurosphere forming assay and Western blot analysis.

### Cell transfection

miR-27b-3p inhibitor and corresponding negative control (NC) were purchased from RiboBio Co., Ltd. (Guangdong, Guangzhou, China). Cells were cultured in a six-well plate before transfection in accordance with the specification of RiboBio using Lipofectamine 3000 reagent (Invitrogen).

MLL4 or PRDM1 was cloned into pcDNA3 expression vectors. shRNA against MLL4 (sh-MLL4) was provided by Genepharma Co. Ltd. (Shanghai, China). The plasmid (0.2 μg) was transfected into cells using lipofectamine 3000 reagents (Invitrogen).

### Neurosphere forming assay and cell viability assessment

GSCs were co-cultured with M2-TAM-derived exosomes (40 μg/mL) for 3 cycles (2 d/cycle). GSCs co-cultured or not with M2-TAM-derived exosomes were dispersed into single cell suspension and seeded in a six-well plate (1 × 10^4^ cells/well) with 0.5% agarose, and cultured in serum-free Dulbecco’s modified Eagle’s medium/Ham’s F-12 medium (DMEM/F12) containing growth factors. After 12 d, the number of spheres in each well was counted. The formation rate of sphere (%) was equal to the number of spheres divided by the number of individual cells initially seeded. The diameter of sphere was measured by Image-Pro Plus 6.0 software. In the light of the manufacturer’s instructions, the determination of cell viability was performed by Cell Titer-Glo Luminescent Cell Viability Assay kit (Promega).

### Isolation and purification of exosomes

TAMs (2.0 × 10^6^ cells/well) were seeded in serum-free high glucose DMEM (Gibco, Carlsbad, California) for 48 h. The medium was centrifuged in a 50 mL centrifuge tube at 300 × *g* for 15 min to collect the supernatant. Next, the supernatant was subjected to a series of low-speed centrifugation steps (2000 × *g*, 10 min) to discard cell debris. Subsequently, the supernatant was centrifuged at 10,000 × *g* for 30 min, ultra-centrifuged at 100,000 × *g* for 120 min (Optima L-100XP, Beckman Coulter) and finally centrifuged for 120 min at 100,000 × *g*. The obtained pellet was the exosomes. The sucrose density gradient fractionation was used for exosome purification [38]. The concentrated exosomes were stored at −80 °C.

### Characterization of exosomes

The morphology of TAM-derived exosomes was observed by a TEM (Tecnai Spirit, FEI). The content of total protein in the exosomes derived from 2.0 × 10^6^ cells within 48 h was measured by bicinchoninic acid (BCA) protein detection kit (23235, Thermo Fisher Scientific, Rochester, NY). TAM-derived exosomes were labeled by PE-bound anti-human CD63 antibody (12-0639-42, Invitrogen), Alexa fluor 488-bound anti-human CD206 antibody (53-2069-42, Invitrogen) and APC-bound anti-human CD163 antibody (17-1639-42, Invitrogen) for flow cytometric analysis. Fluorescence imaging was performed by incubation with rabbit anti-CD206 antibody (1:50, ab64693, Abcam, Cambridge, UK), rabbit anti-CD163 antibody (1:60, ab182422, Abcam) and rabbit anti-CD63 antibody (1:50, ab59479, Abcam) at 4 °C overnight, and incubation with Alexa fluor 488 bound anti-rabbit antibody (1:200, ab15008, Abcam) for 1 h. The images were finally captured under a fluorescence confocal microscope (A1, Nikon, Tokyo, Japan). Rabbit anti-CD206 antibody (1:1000, ab64693, Abcam), rabbit anti-CD163 antibody (1:1000, ab182422, Abcam) and rabbit anti-CD63 antibody (1:1000, ab216130, Abcam) were used for Western blot analysis.

### Uptake of exosomes

Exosomes were labeled with 10 μL CM Dil dye (Sigma-Aldrich Chemical Company, St Louis MO) for 5 min at 37 °C and then incubated for 15 min at 4 °C. After fluorescence labeling, exosomes were rinsed three times with PBS and suspended in PBS. GSCs were co-cultured with 20 μg/mL exosomes for 6 h and observed under a confocal laser scanning microscope (CLSM) (TCS SP5-II, Leica Microsystems, Nanterre, France).

### RT-qPCR

Total RNA was extracted using TRIzol reagent (15596026, Invitrogen). In accordance with the specification of PrimeScript RT reagent Kit (RR047A, Takara, Kyoto, Japan), mRNA was reversely transcribed into complementary DNA (cDNA), while PolyA Tailing Reverse Transcription Kit (B532451-0010, Shanghai Sangon Biotechnology Co. Ltd., Shanghai, China) was applied for miRNA reverse transcription into cDNA. Next, RT-qPCR was conducted using Fast SYBR Green PCR kit (Applied Biosystems, Carlsbad, CA) in ABI PRISM 7300 RT-PCR system (Applied Biosystems). β-actin and U6 served as the loading controls of genes and miRNAs, respectively. The relative expression of RNA was analyzed using the 2^−ΔΔCt^ method. The primers are shown in Supplementary Table 1.

### Western blot analysis

Cells and exosomes were lysed by RIPA lysis buffer, and the total protein was extracted. The denatured protein sample (20 μg) was separated by 12% SDS-PAGE and transferred to the polyvinylidene fluoride membrane. The membrane was blocked with 5% skimmed milk and probed with primary antibodies (Abcam) against CD133 (1:1000, ab19898), Nestin (Mouse, 1:2000, ab254048), GFAP (1:10,000, ab7260), Oct4 (1:1000, ab181557), Sox2 (1:1000, ab92494), MLL4 (Mouse, 1:1000, ab56770), H3K4me1 (1:5000, ab176877), H3K9me3 (1:1000, ab8898) and β-actin (1:5000, ab179467). The peroxidase-labeled goat anti-rabbit or anti-mouse IgG (H+L) served as secondary antibody. The blots were developed by chemiluminescence system (Millipore, Billerica, MA). The density of western blot band was quantified by ImageJ software, and normalized to β-actin.

### Tumor xenograft in NOD/SCID mice

An in-situ orthotopic xenograft model was developed in mice by intracranial injection of GBM cells [39]. Male non-obese diabetic/severe combined immunodeficiency (NOD/SCID) mice (4–6 weeks old) were purchased from Guangzhou Saiye Biotechnology Co., Ltd. (Guangzhou, China). In order to discuss the tumor-promoting effects of TAM-derived exosomes on GSC-driven tumor growth, TAM-derived exosomes and GSCs (5 × 10^3^ cells per mouse) were transplanted into the brain of mice.

### Histological analyses

After 40 days, the brain of mice bearing GBM was fixed by 10% neutral formalin solution and sliced into sections. The tissue sections were stained with HE and sealed by neutral gum. The histopathological changes of tissues were observed under the BX51 microscope (Olympus Optical Co. Ltd, Tokyo, Japan). For immunohistochemical staining, the primary antibody anti-Sox2 (#3579, 1: 200, Cell Signaling Technology, Beverly, MA) was employed. The percentage of Sox2 positive cells was quantified in five randomly selected regions of each tumor specimen.

### Dual-luciferase reporter assay

MLL4 3′UTR was amplified and cloned into a dual-luciferase reporter vector (Promega, Madison, WI) and named as psiCHECK2-MLL4-WT. A mutated MLL4 3′UTR reporter plasmid (psiCHECK2-MLL4-MUT) was produced by mutating the miR-27b-3p binding region. In order to evaluate the direct binding between miR-27b-3p and MLL4 3′UTR, HEK-293 cells were co-transfected with 100 nM miR-27b-3p mimic and 1 μg psiCHECK2-MLL4-WT or psiCHECK2-MLL4-MUT. At 48 h post-transfection, the luciferase activity was determined by Dual-Glo luciferase assay system (Promega).

### ChIP assay

ChIP kit (Millipore) was adopted to test the binding of PRDM1 enhancer to IL-33 promoter. Upon reaching about 70–80% confluence, cells were cross-linked with 1% formaldehyde for 10 min and then randomly broken into fragments by ultrasonication. The supernatant was collected after centrifugation at 13,000 rpm and 4 °C. The supernatant was severally incubated with positive control antibody RNA polymerase II, anti-human IgG and rabbit anti-H3K4me1 (1:100, ab176877, Abcam) and H3K9me3 (1:100, ab8898, Abcam) at 4 °C overnight. Endogenous DNA-protein complex was precipitated by Protein Agarose/Sepharose and centrifuged to remove the supernatant, and nonspecific complex was rinsed. The cross-linking was reversed at 65 °C overnight. DNA fragments were purified and recovered by phenol/chloroform extraction. The expression of PRDM1 enhancer and IL-33 promoter was determined by RT-qPCR.

### Statistical analysis

All data were analyzed by SPSS 21.0 software (IBM Corp. Armonk, NY). Measurement data were indicated as mean ± standard deviation. Comparison between two groups was conducted by unpaired *t* test, while that among multiple groups by one-way analysis of variance (ANOVA) followed by Tukey’s post hoc test. The data at different time points were compared using repeated measures ANOVA with Bonferroni’s post hoc test. *p* value less than 0.05 was indicative of statistically significant difference.

## Supplementary information

Supplementary Figure 1

Supplementary Table 1

Supplemental Material

## Data Availability

The datasets generated/analyzed during the current study are available.
