# Supplementary material for: M2-like tumor-associated macrophages transmit exosomal miR-27b-3p and maintain glioblastoma stem-like cell properties
Source: Cell Death Discov. 2022 Aug 4;8:350. doi: 10.1038/s41420-022-01081-7 (PMC9352681; doi:10.1038/s41420-022-01081-7)
Supplement: Supplementary file 1 — Supplementary Figure 1 [file 41420_2022_1081_MOESM1_ESM.doc]

**
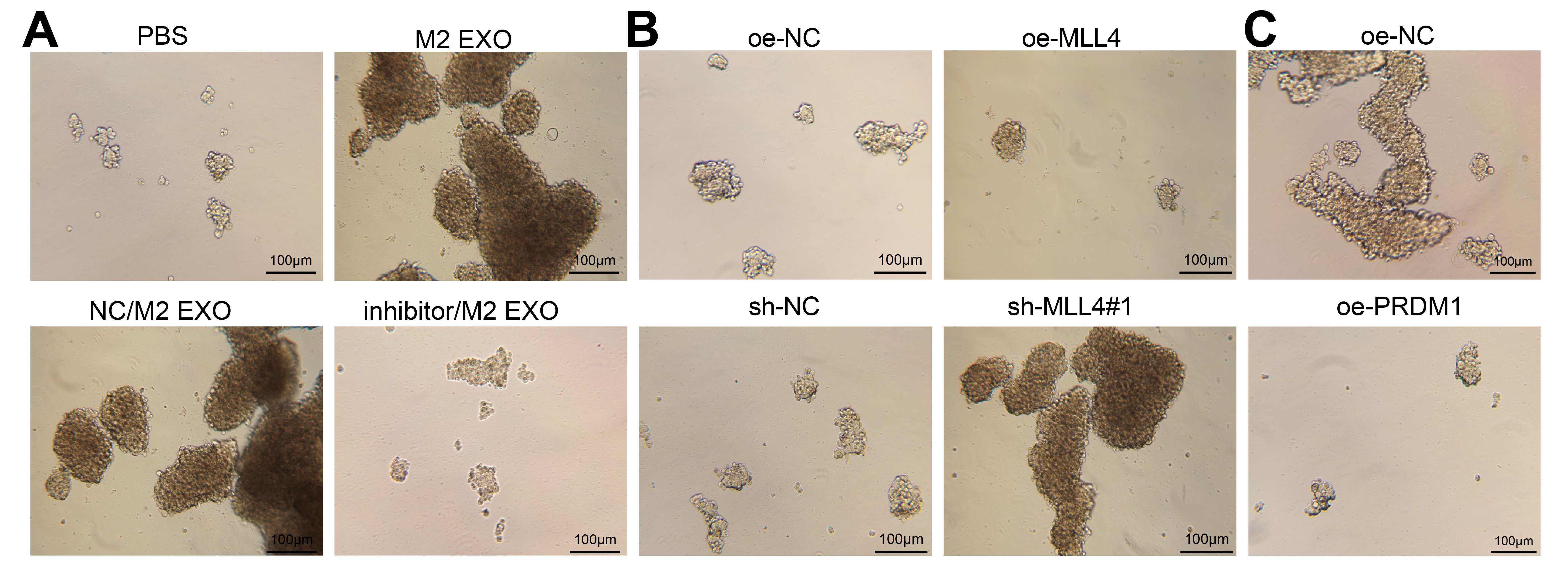
**

**Supplementary Figure 1** Representative images of neurosphere formation. A, The formation rate and diameter of spheres in GSCs in response to M2-TAM-derived exosomes alone or miR-27b-3p inhibitor in the M2-TAM-derived exosomes. B, The formation rate and diameter of spheres in GSCs in response to oe-MLL4 or sh-MLL4#1. C, The formation rate and diameter of spheres in GSCs in response to oe-PRDM1.
