# Supplementary material for: M2-like tumor-associated macrophages transmit exosomal miR-27b-3p and maintain glioblastoma stem-like cell properties
Source: Cell Death Discov. 2022 Aug 4;8:350. doi: 10.1038/s41420-022-01081-7 (PMC9352681; doi:10.1038/s41420-022-01081-7)
Supplement: Supplementary file 2 — Supplementary Table 1 [file 41420_2022_1081_MOESM2_ESM.doc]

**Supplementary Table 1** PCR primer sequences

| Gene | Sequence |
| --- | --- |
| CD163 | F: 5’-TTTGTCAACTTGAGTCCCTTCAC-3’ |
|  | R: 5’-TCCCGCTACACTTGTTTTCAC-3’ |
| miR-27b-3p | F: 5’-GCGCGTTCACAGTGGCTAAG-3’ |
|  | R: Universal reverse primer |
| IL-33 | F: 5’-GTGACGGTGTTGATGGTAAGAT-3’ |
|  | R: 5’-AGCTCCACAGAGTGTTCCTTG-3’ |
| PRDM1 | F: 5’-AAGCAACTGGATGCGCTATGT-3’ |
|  | R: 5’-GGGATGGGCTTAATGGTGTAGAA-3’ |
| MLL4 | F: 5’-GGGTCGCAAGCATAAGACGA-3’ |
|  | R: 5’-CATCCGTTCTGTGCCTTCCT-3’ |
| U6 | F: 5’-ATTGGAA CGATACAGAGAAGATT-3’ |
|  | R: Universal reverse primer |
| β-actin | F: 5’-AGG GGCCGGACTCGTCATACT-3’ |
|  | R: 5’-GGCGGCAC CACCATGTACCCT-3’ |

Note: PCR, polymerase chain reaction; F, forward; R, reverse; miR-27b-3p, microRNA-27b-3p; IL-33, interleukin-33; PRDM1, positive regulatory domain I; MLL4, mixed-lineage leukemia 2.
