# Supplementary material for: M2-like tumor-associated macrophages transmit exosomal miR-27b-3p and maintain glioblastoma stem-like cell properties
Source: Cell Death Discov. 2022 Aug 4;8:350. doi: 10.1038/s41420-022-01081-7 (PMC9352681; doi:10.1038/s41420-022-01081-7)

**Supplemental Material - original western blots**

Figure 1G


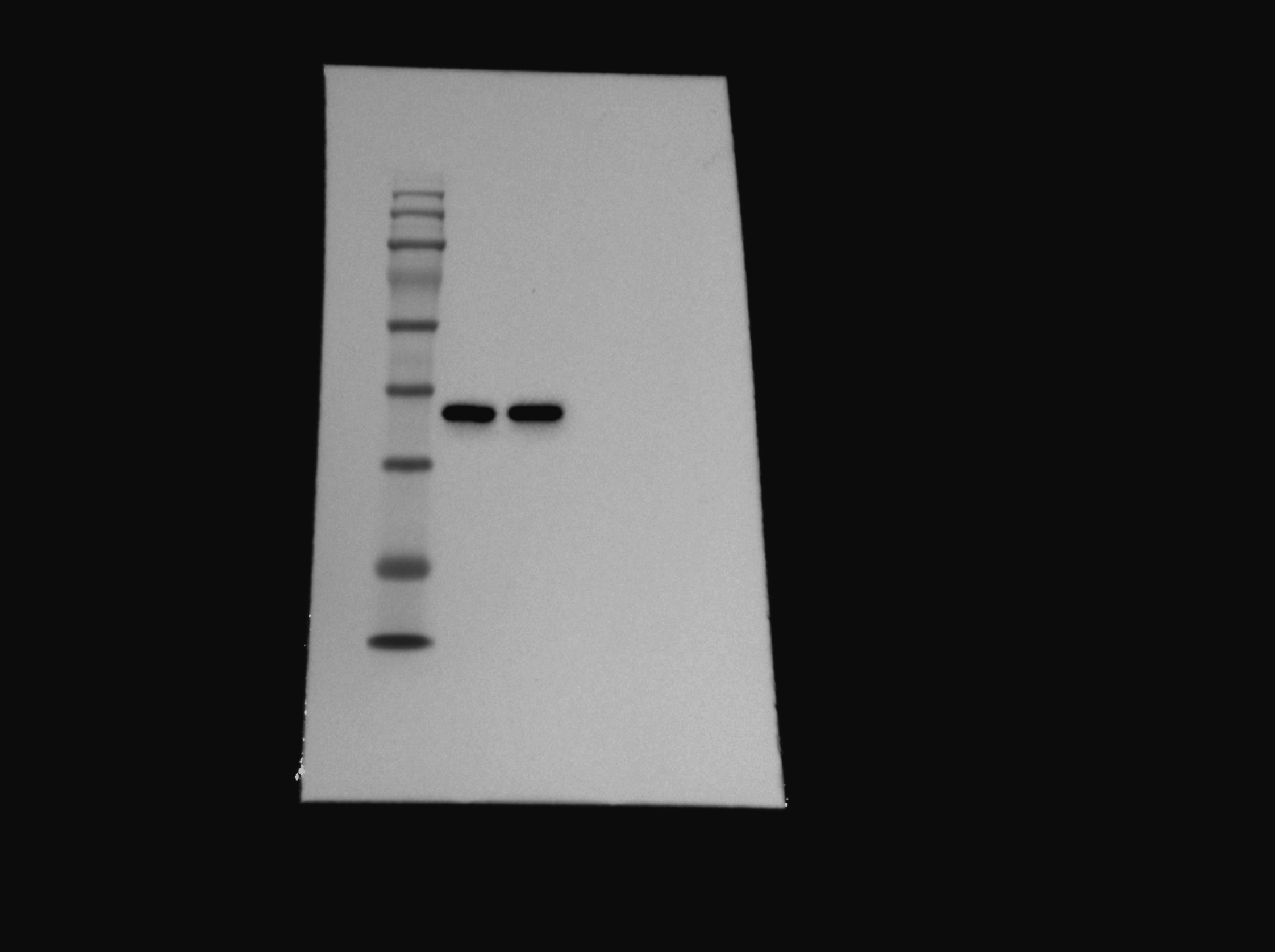

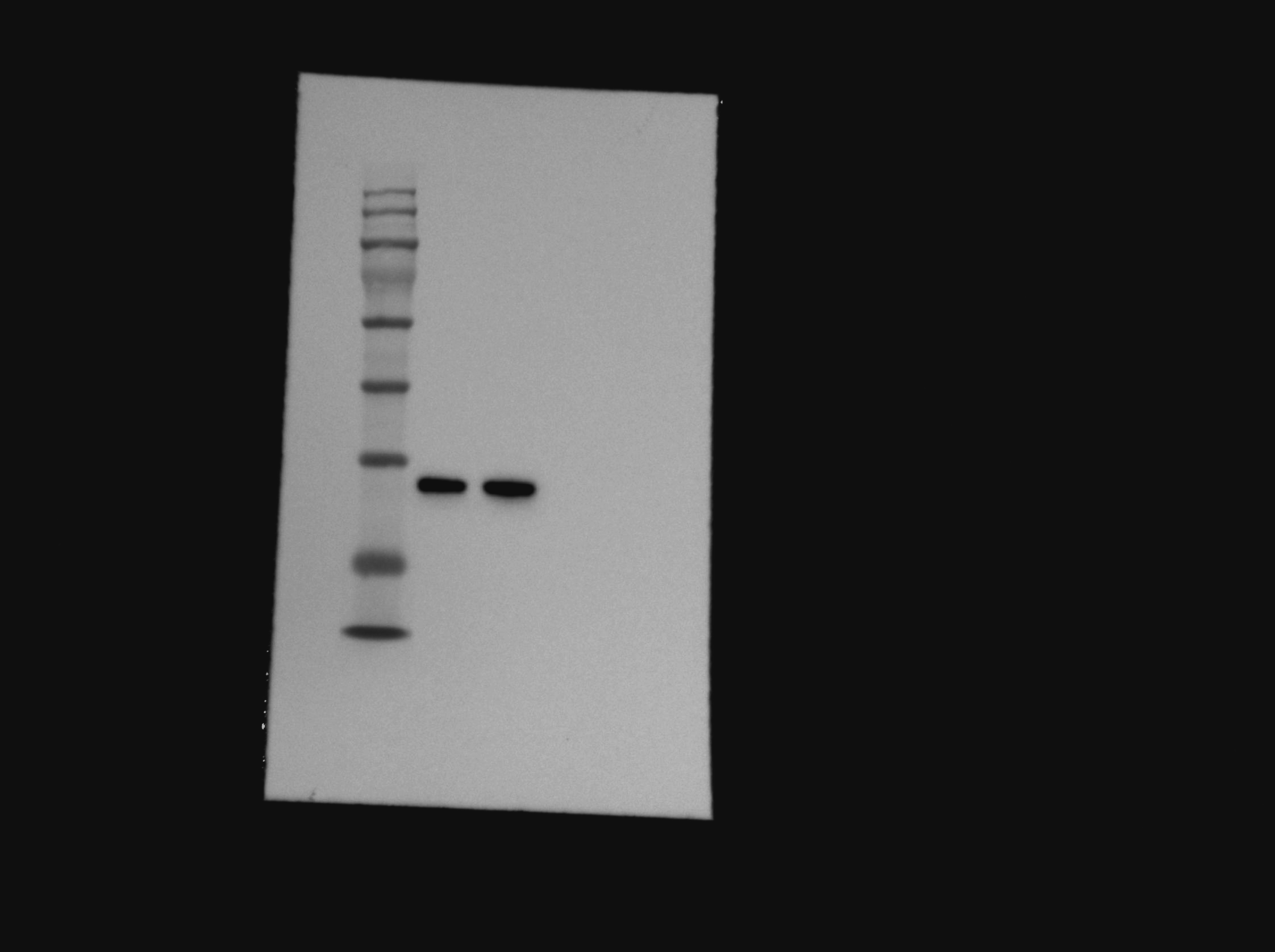

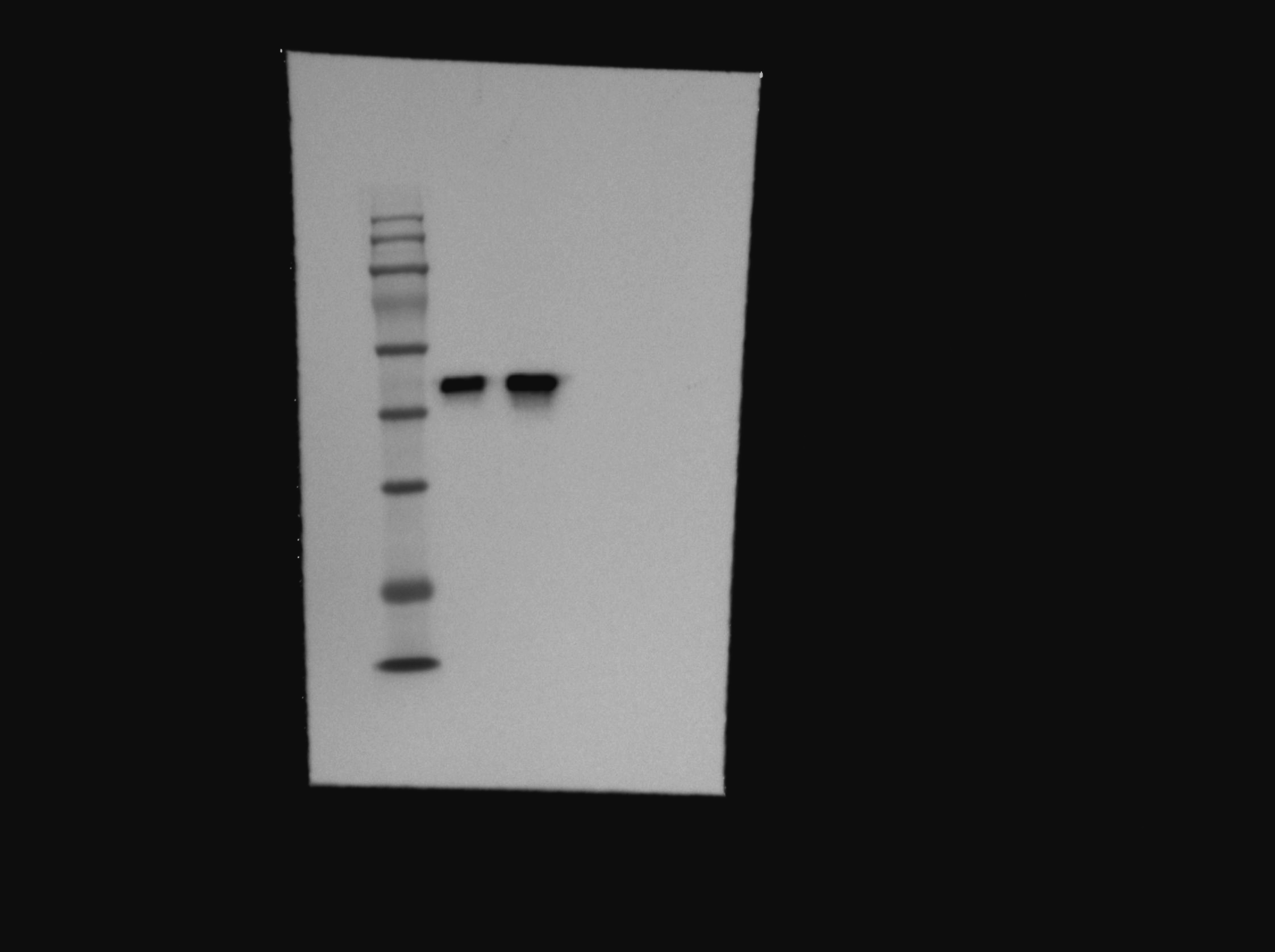

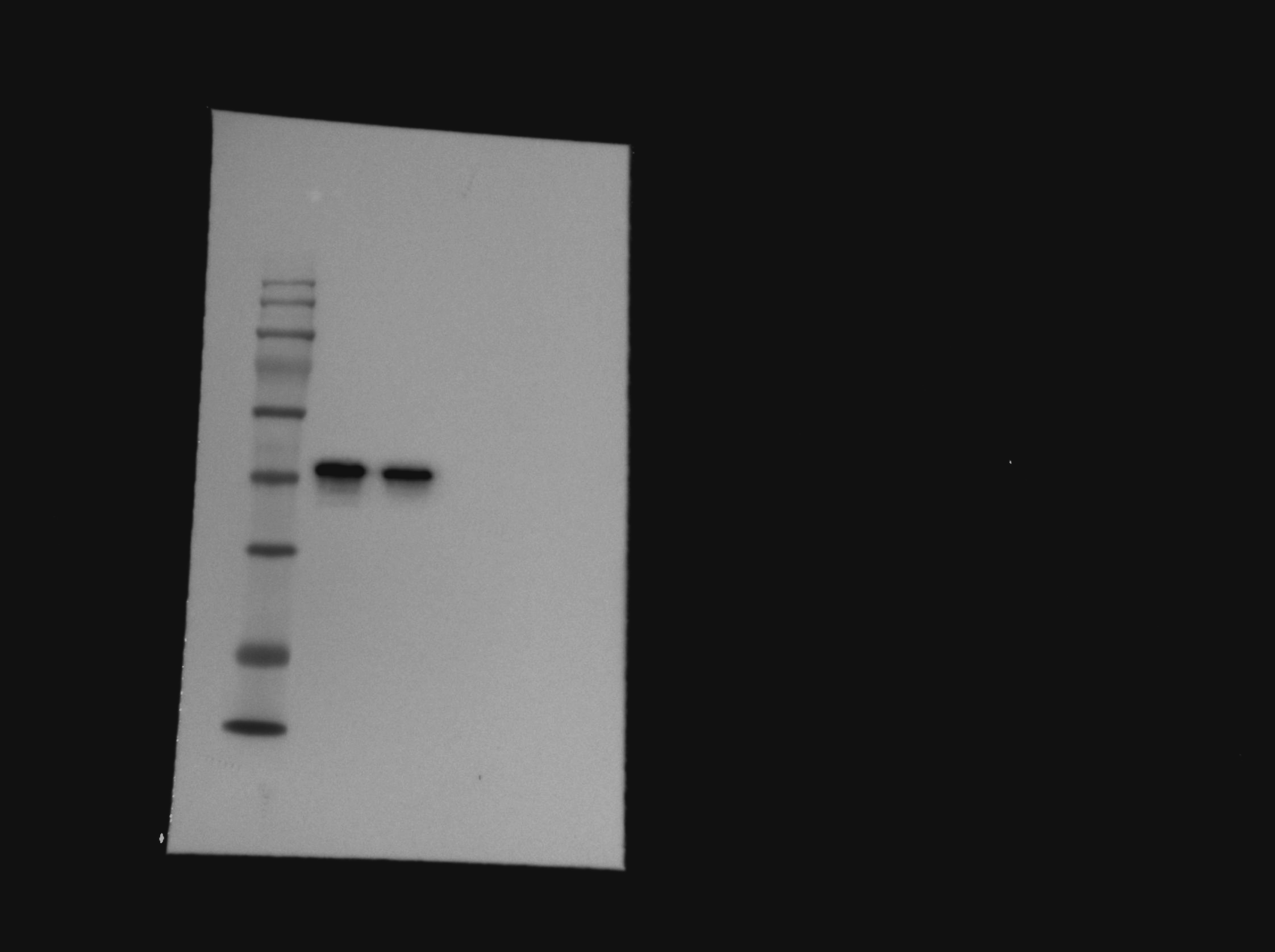

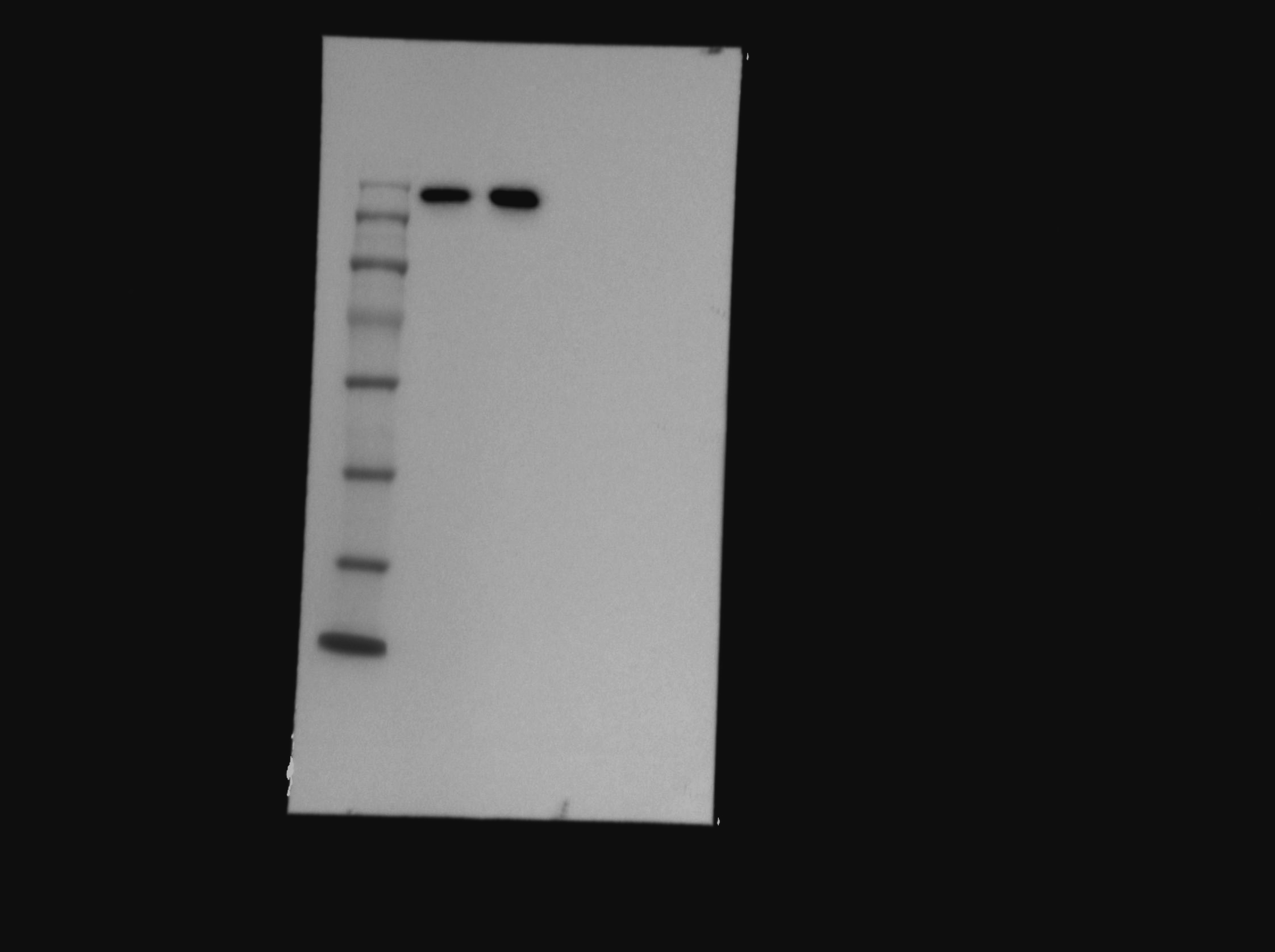

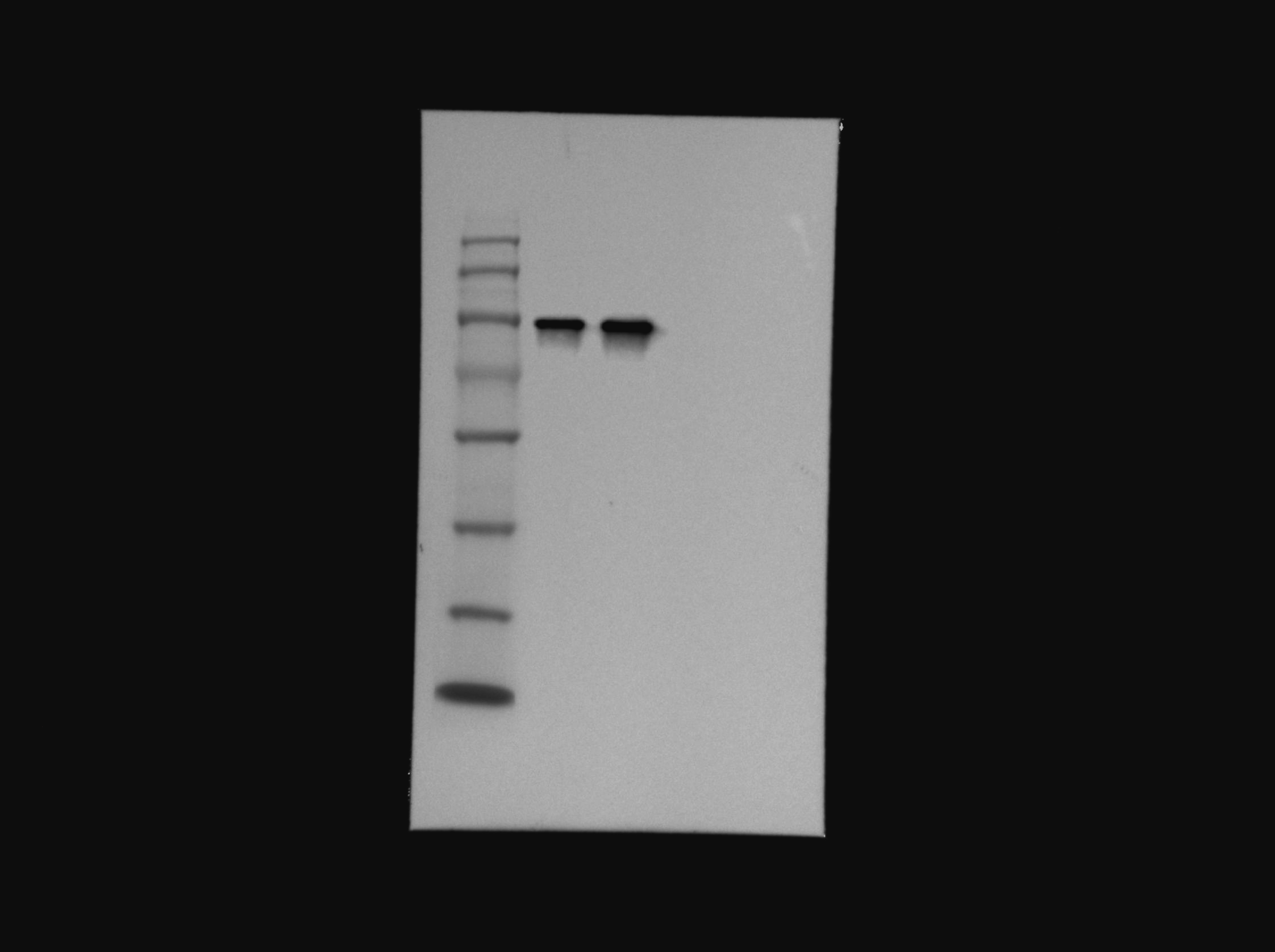


Figure 2C


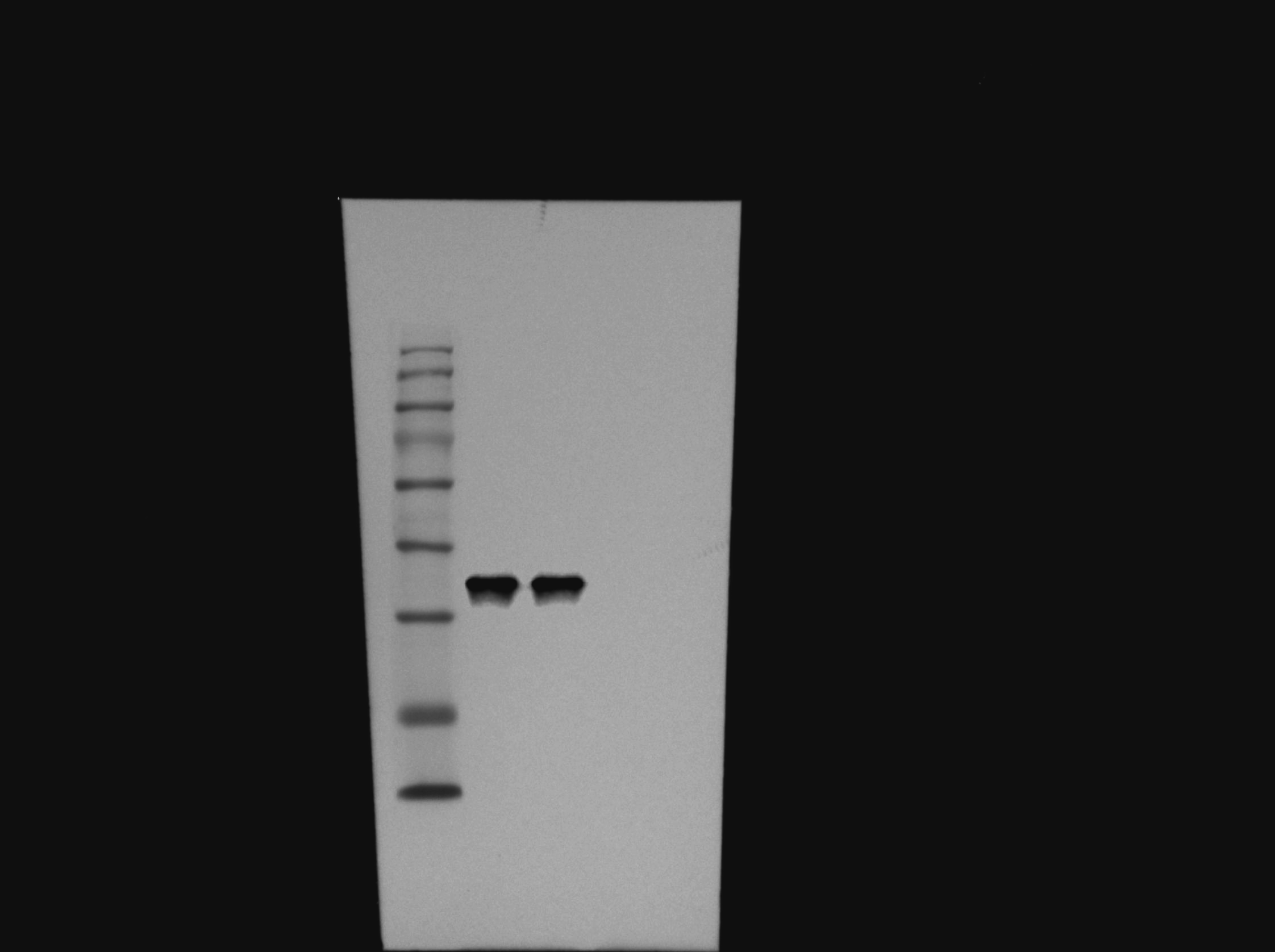

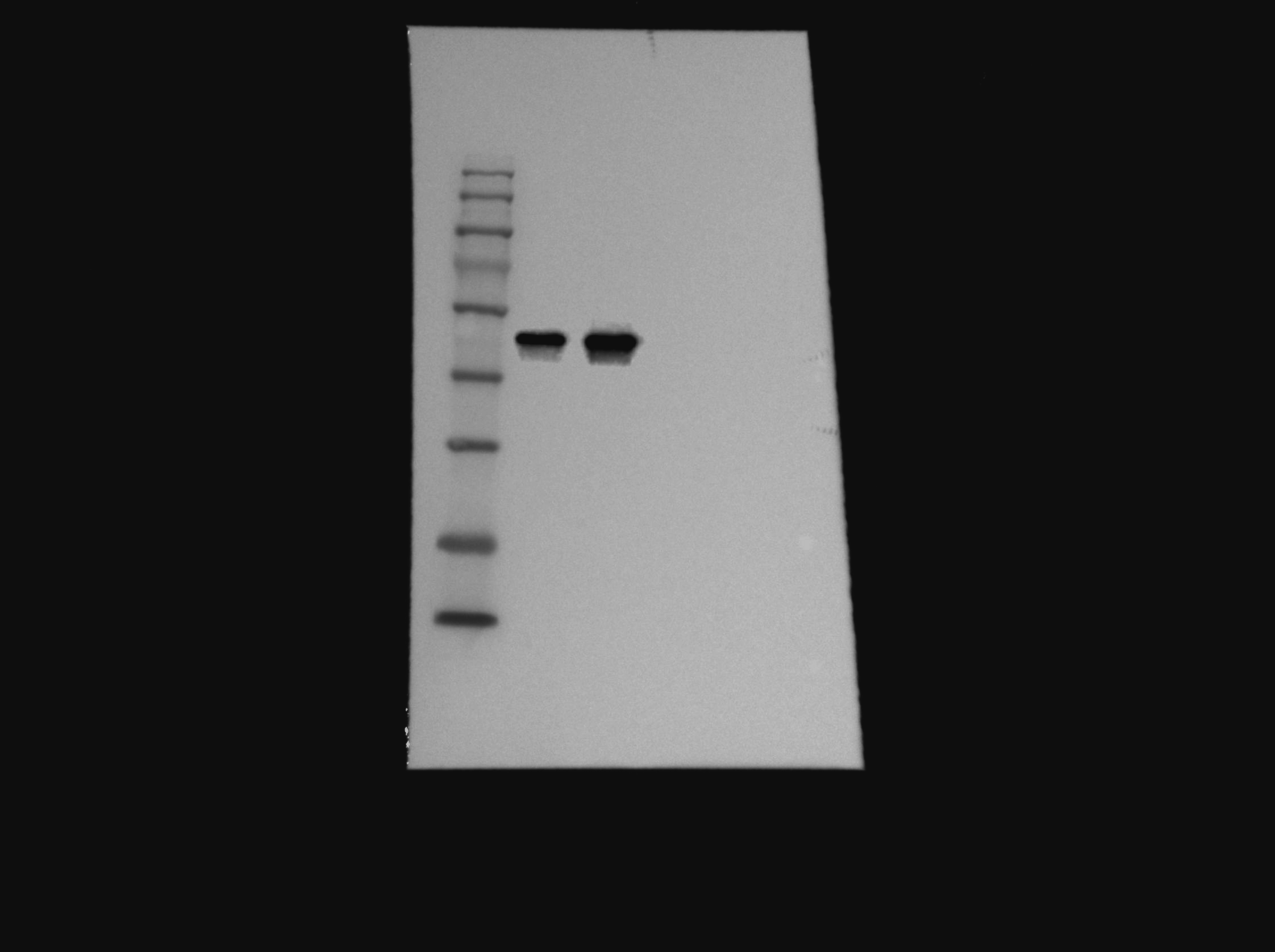

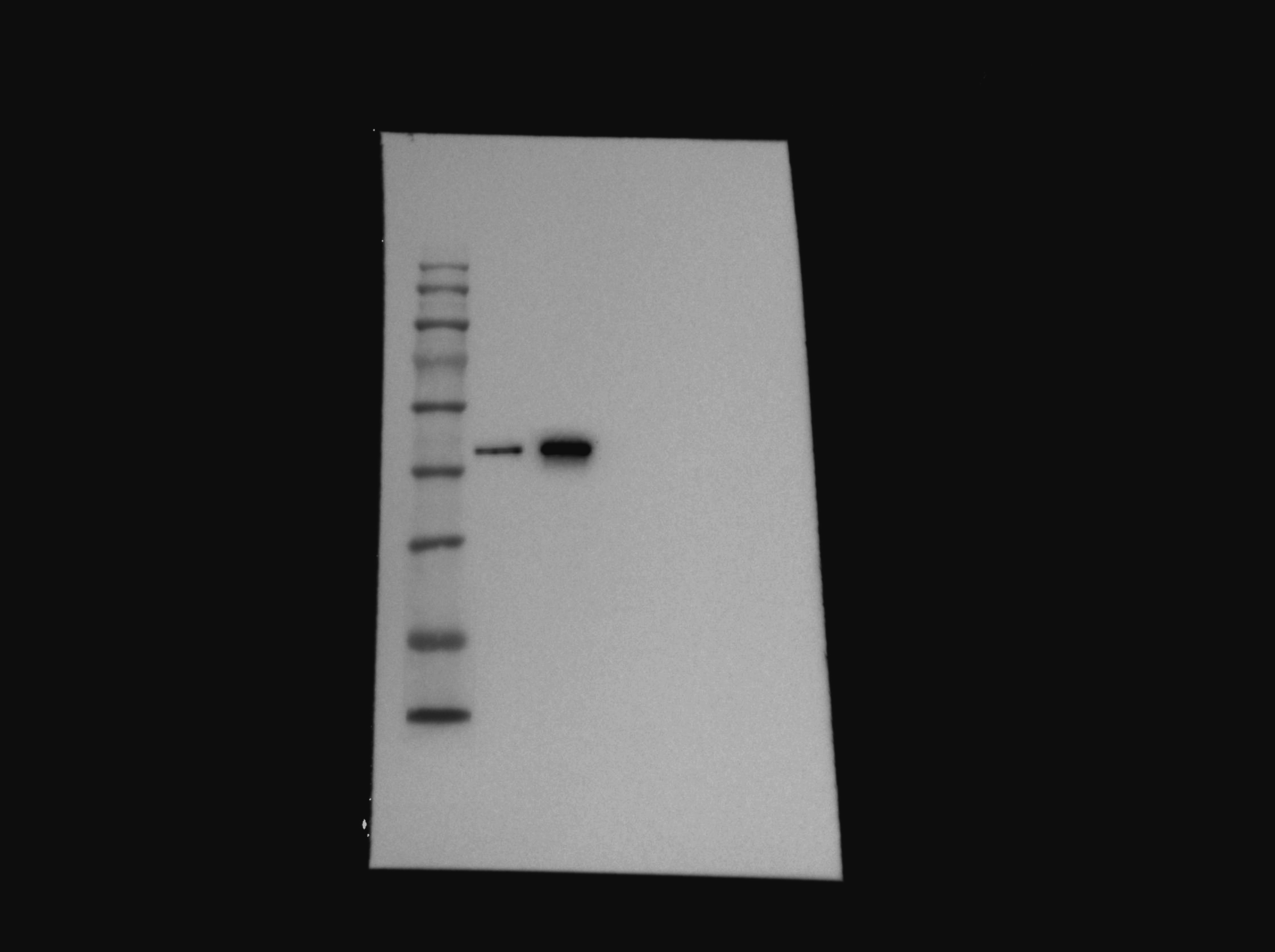

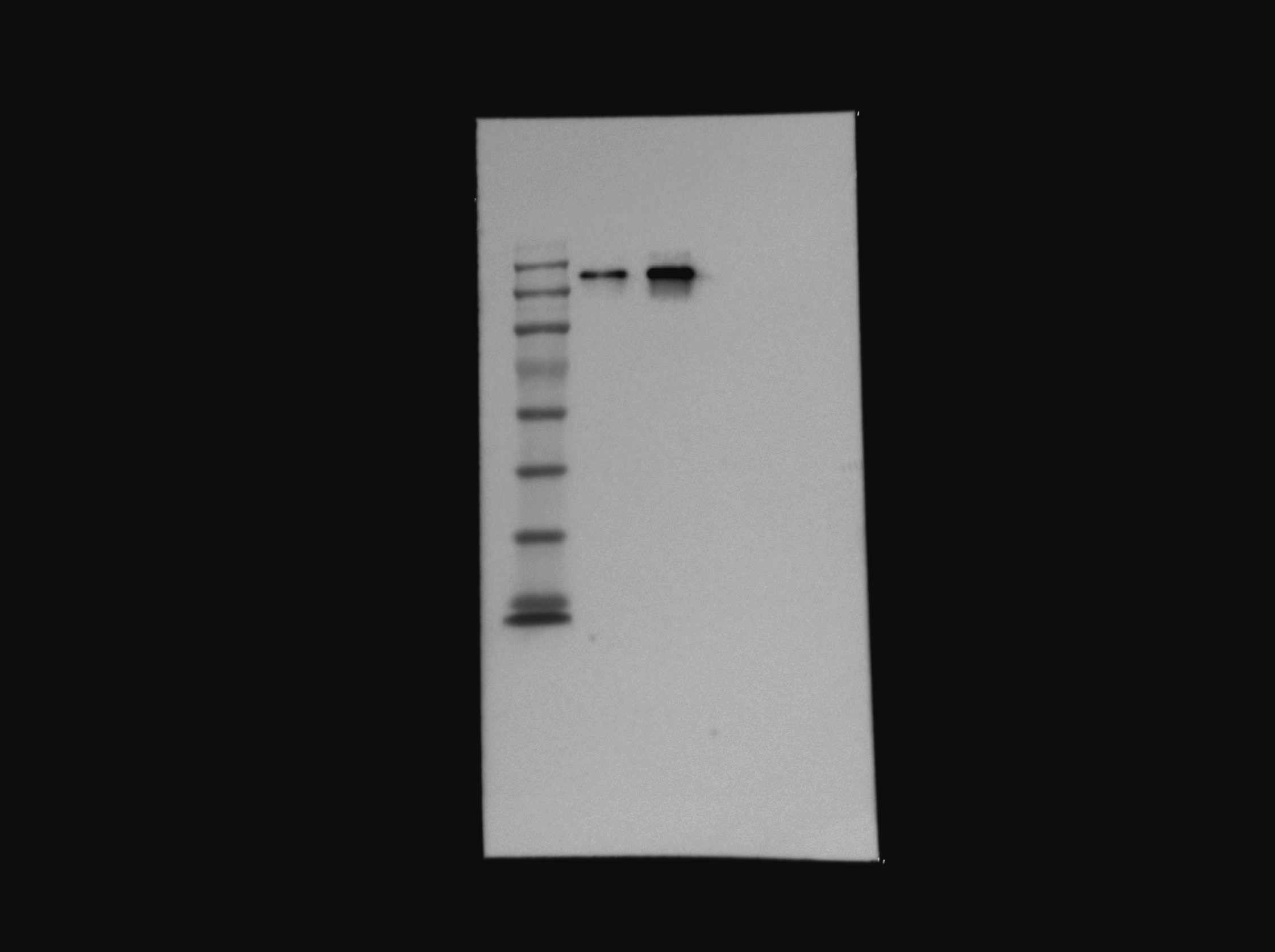

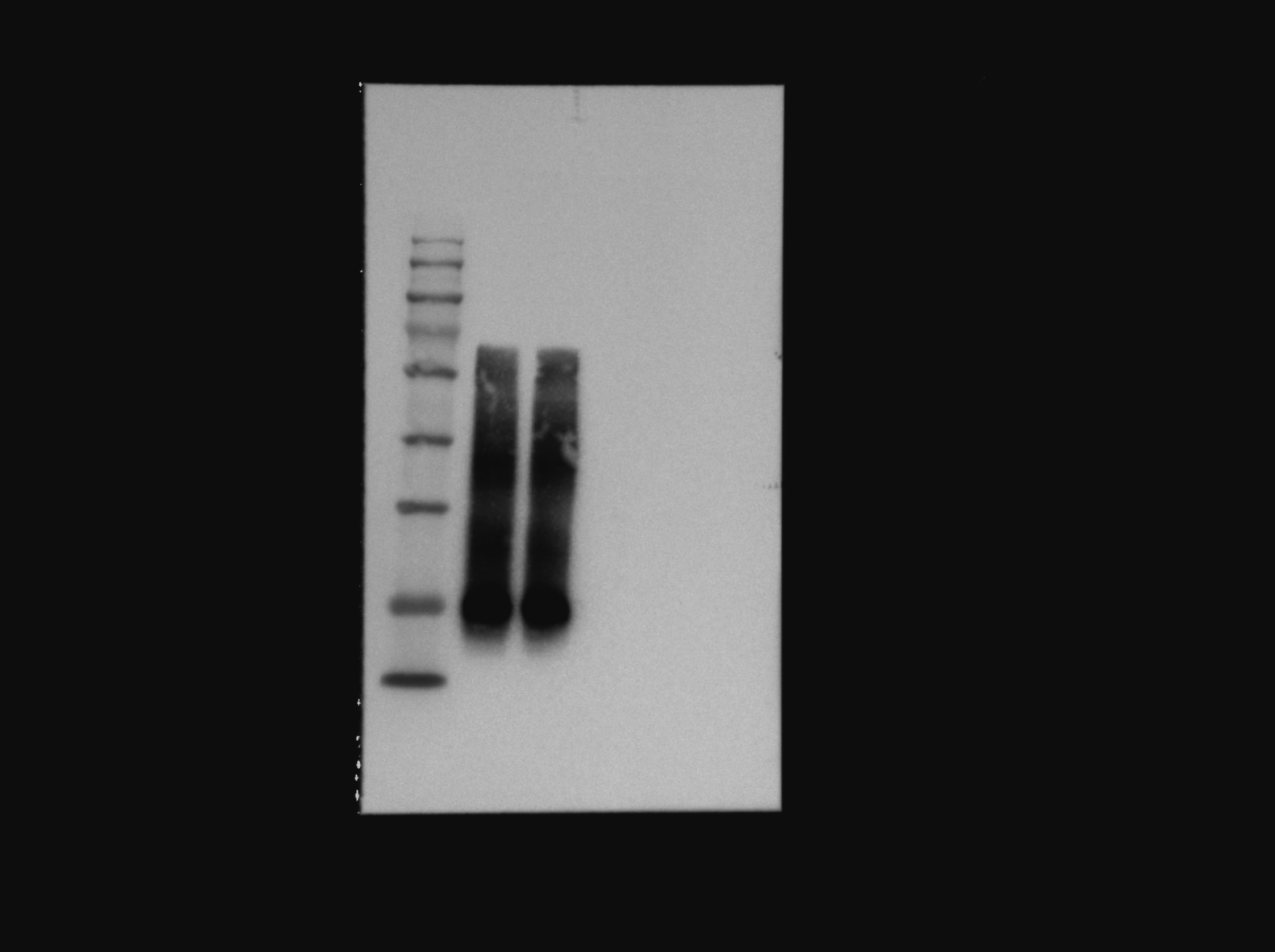


Figure 4D


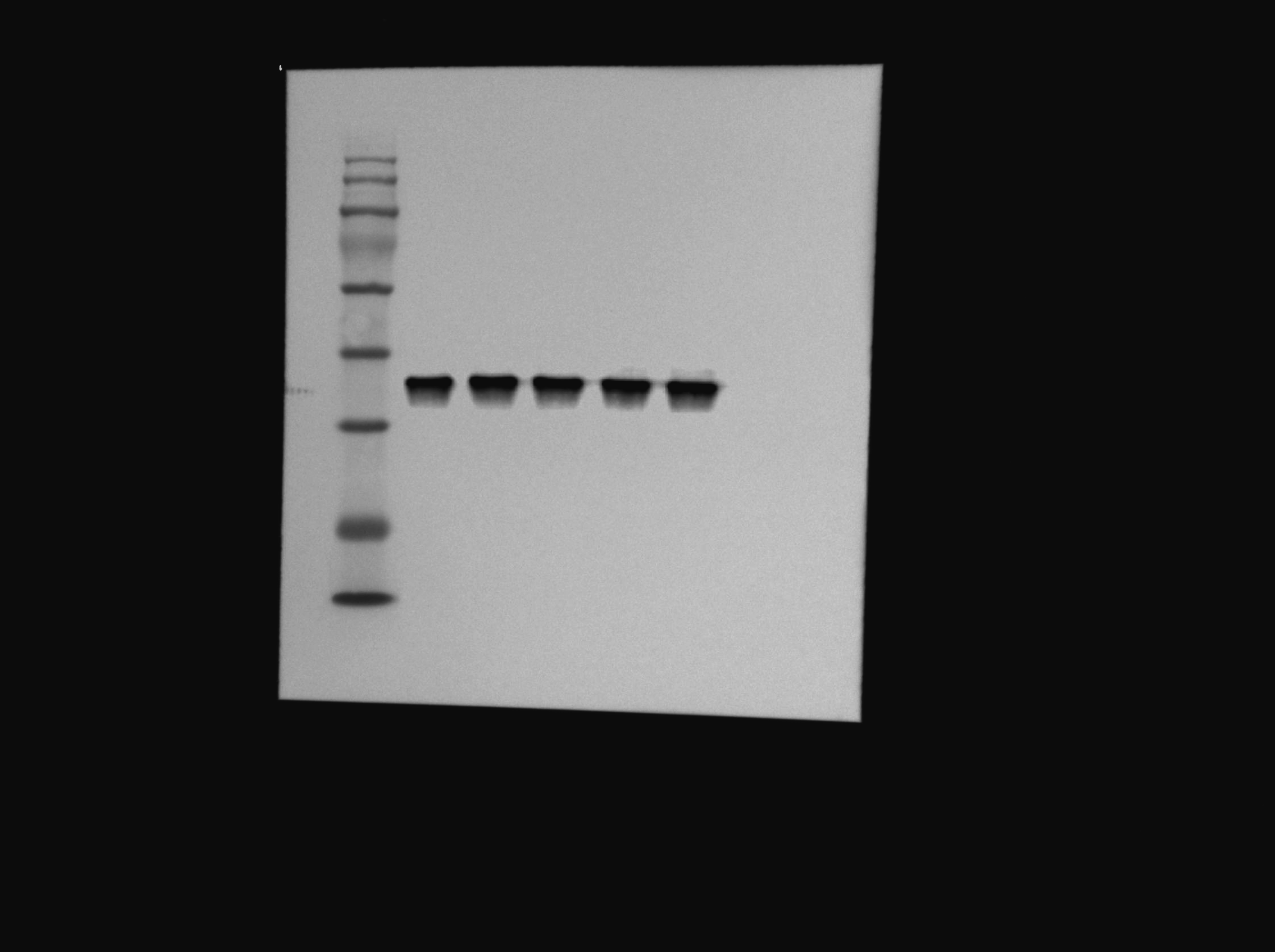

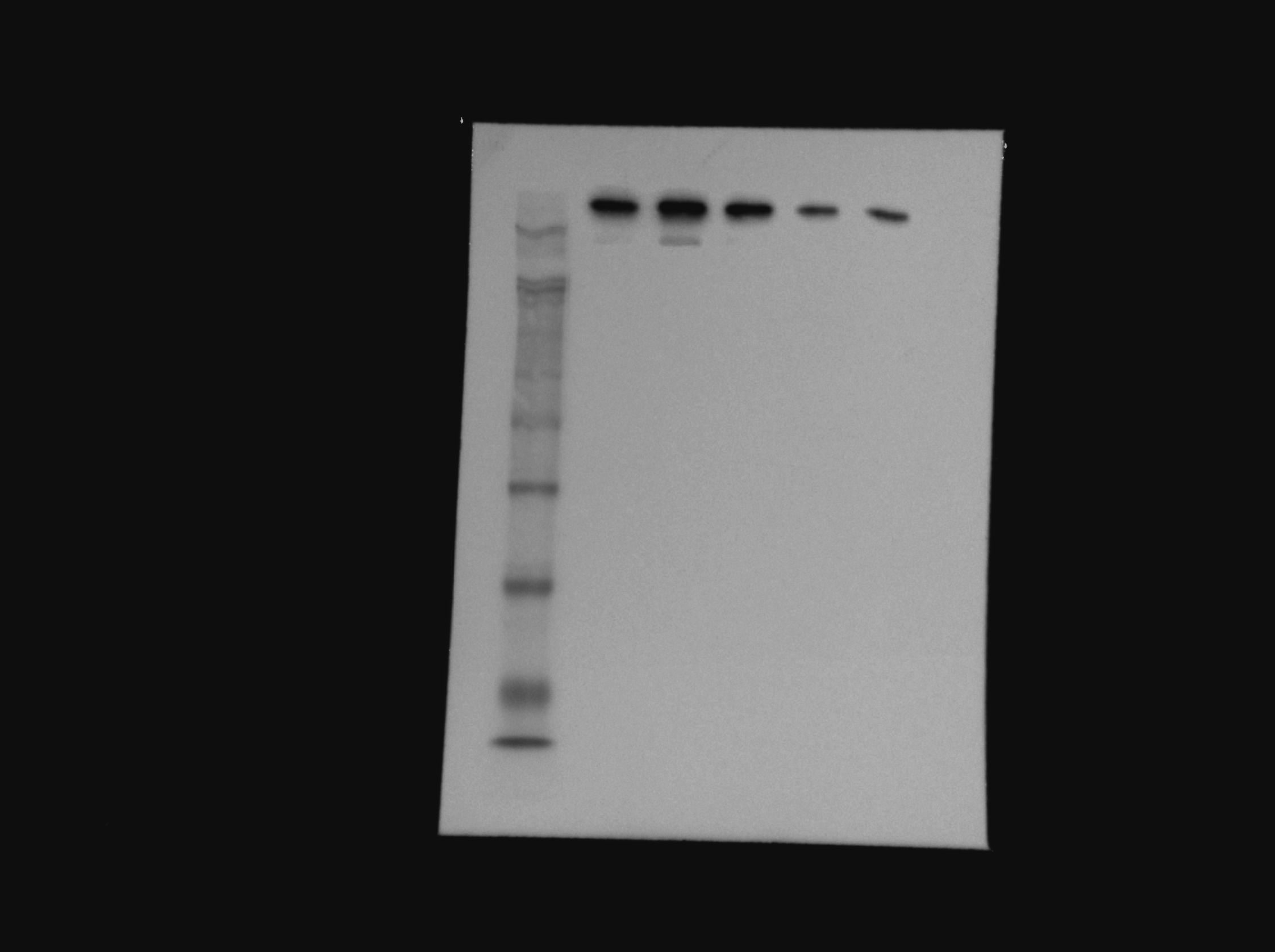


Figure 5C


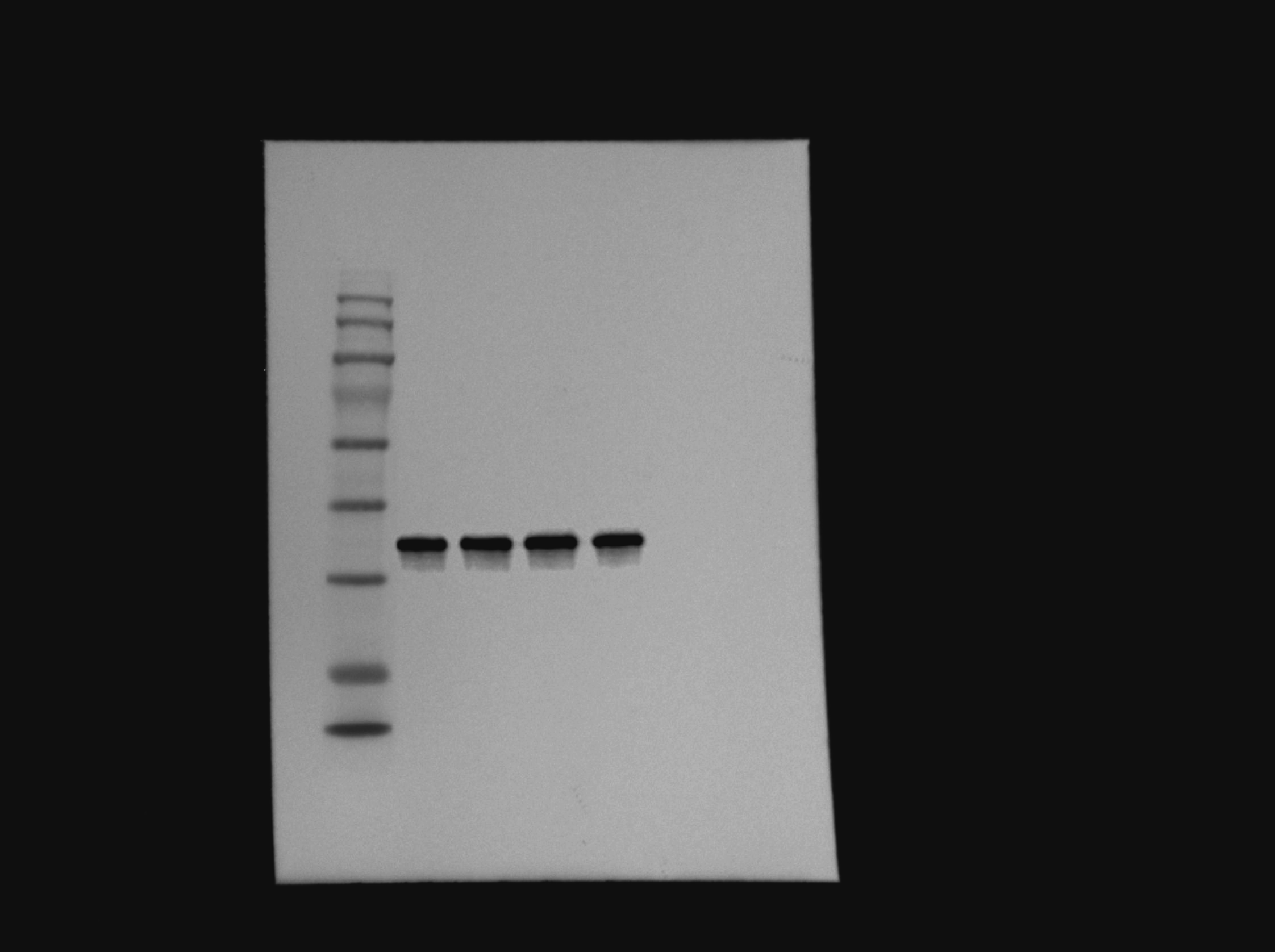

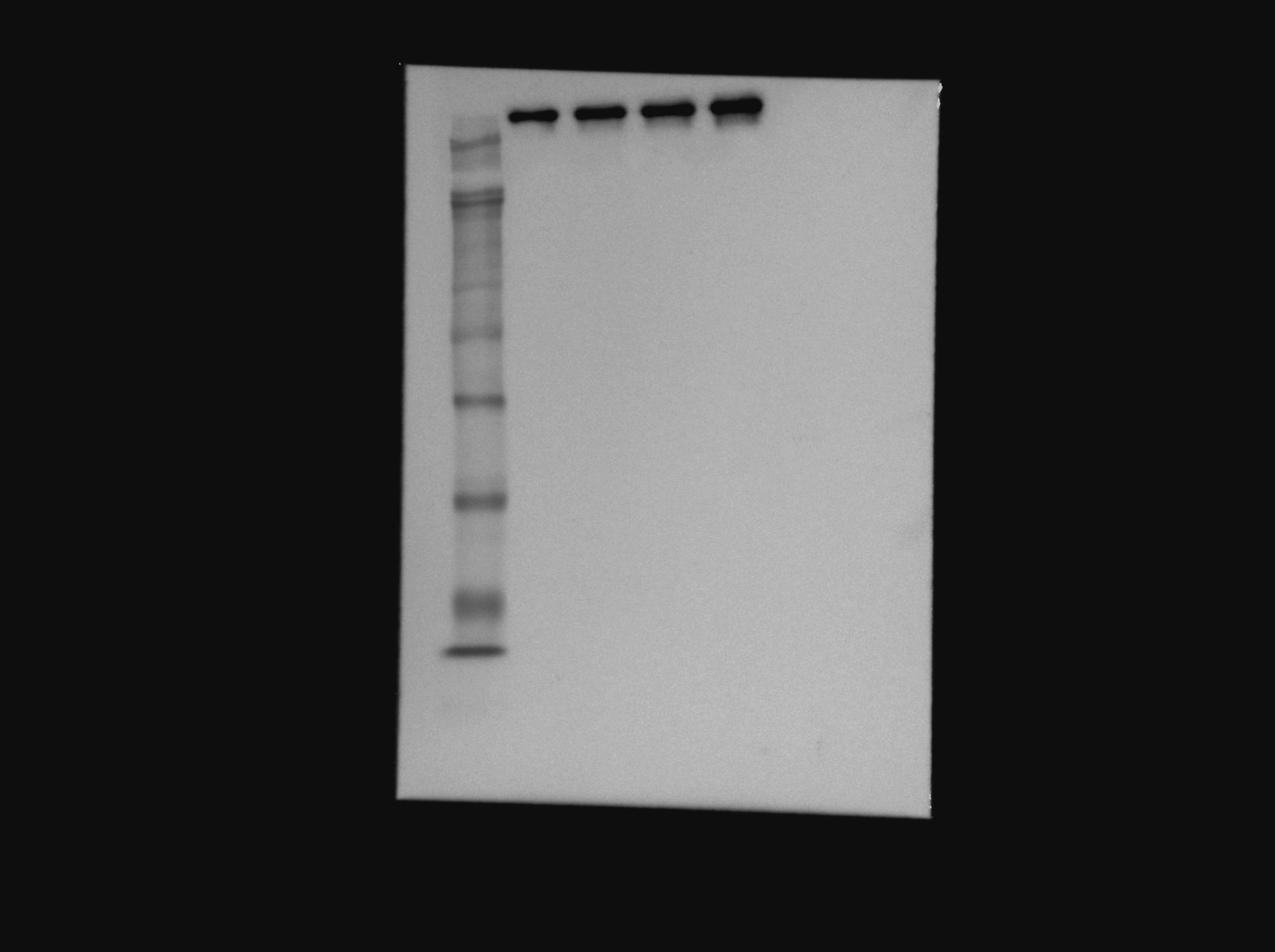


Figure 5E


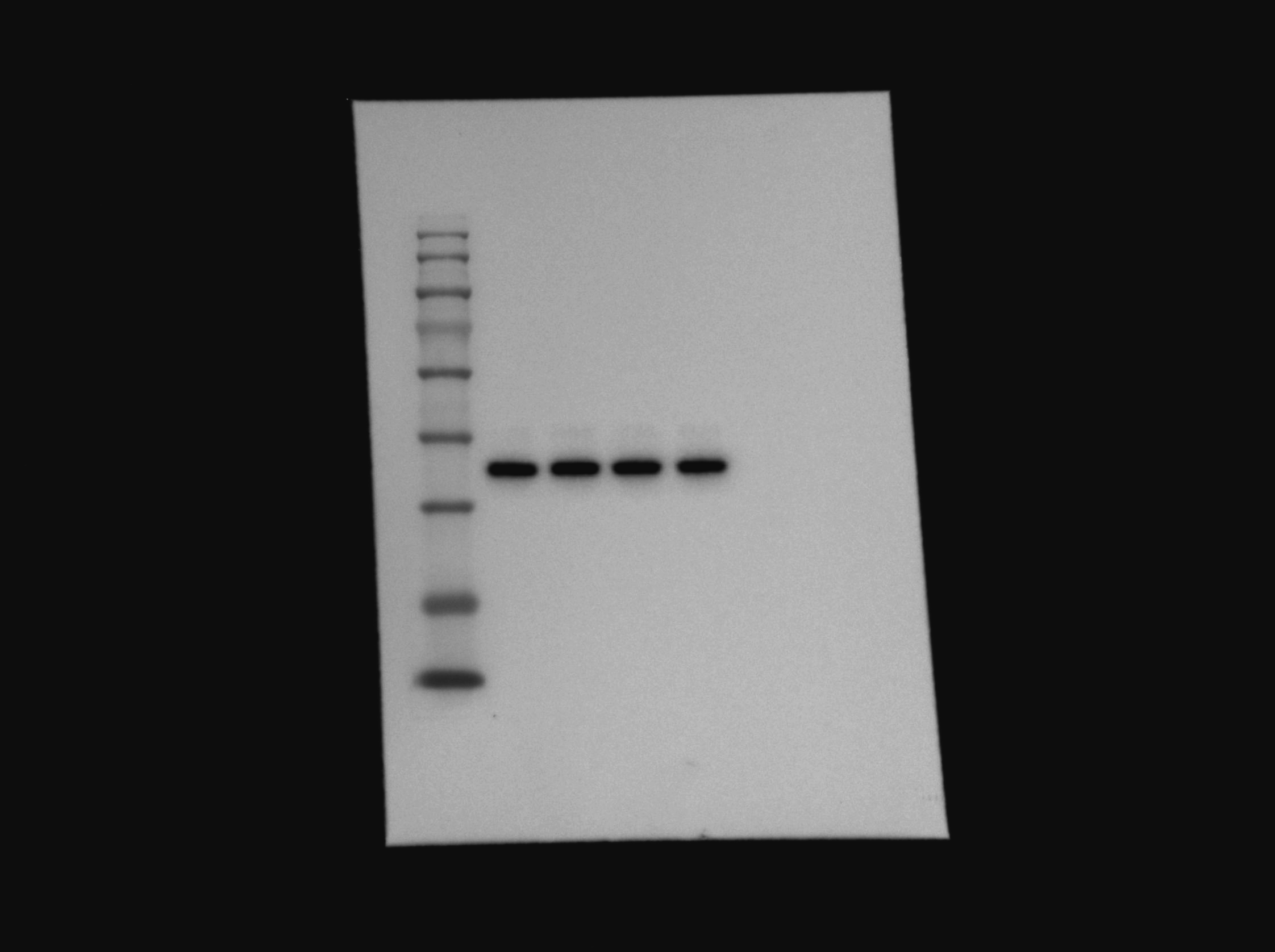

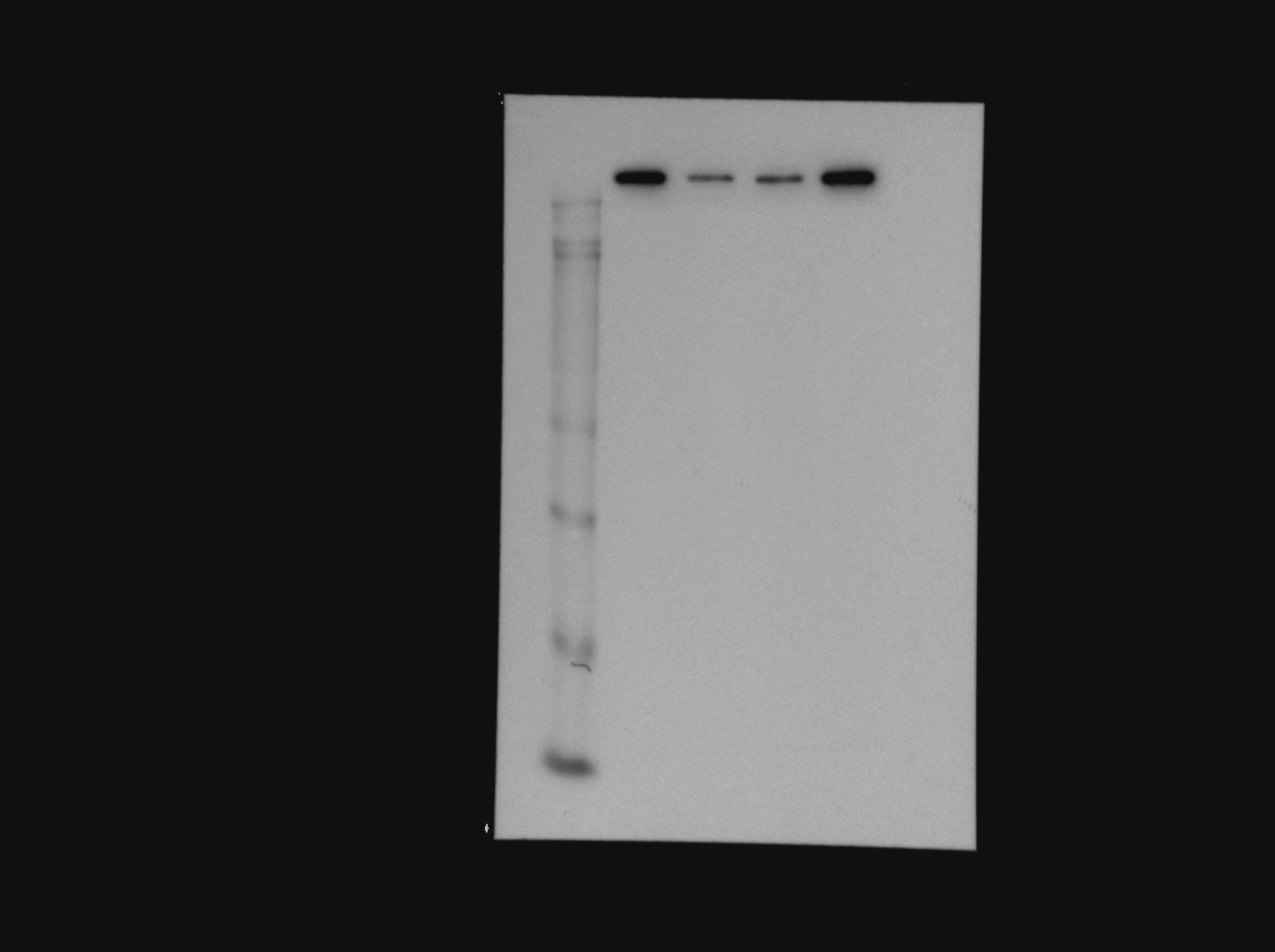


Figure 6G


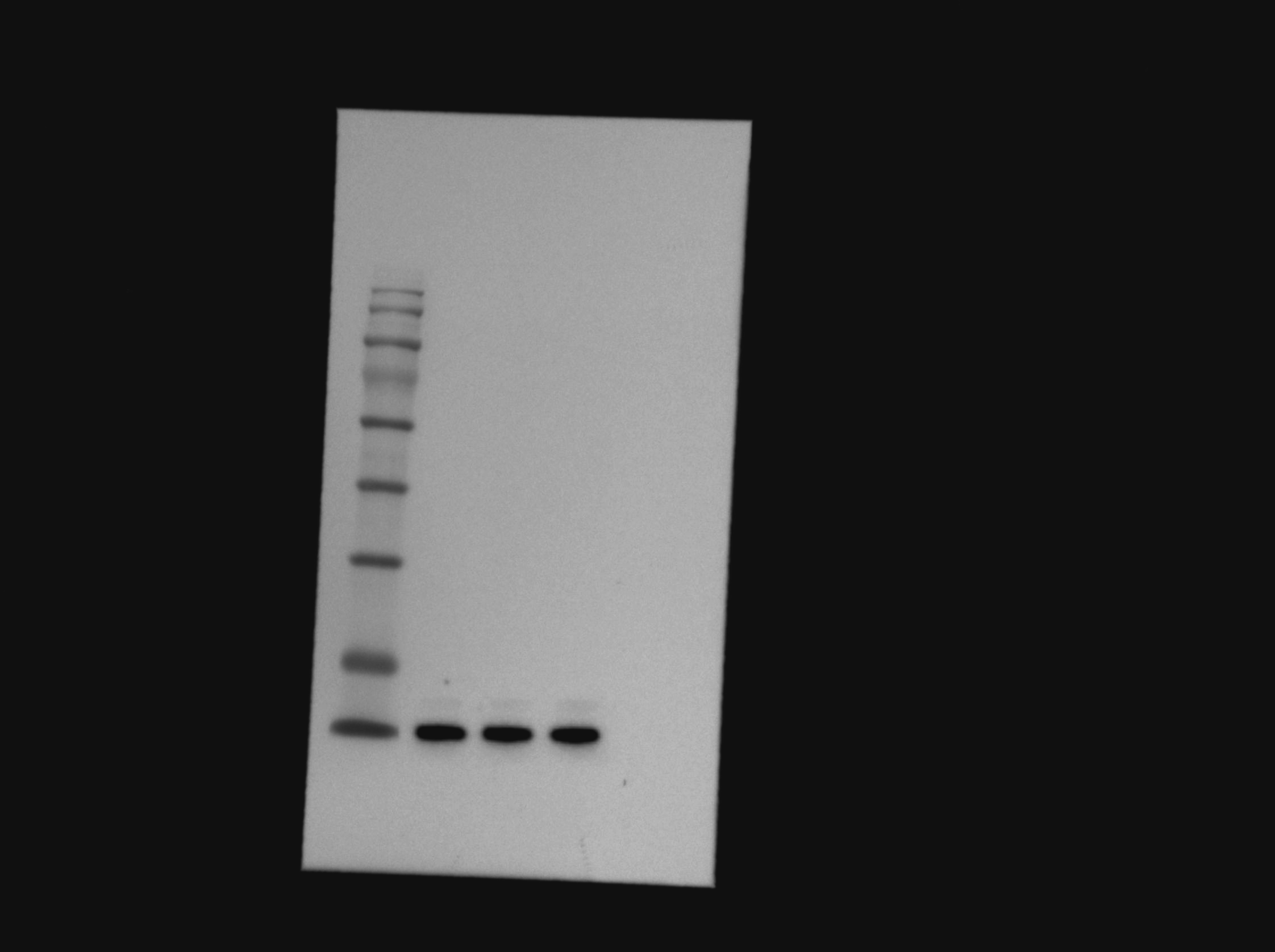

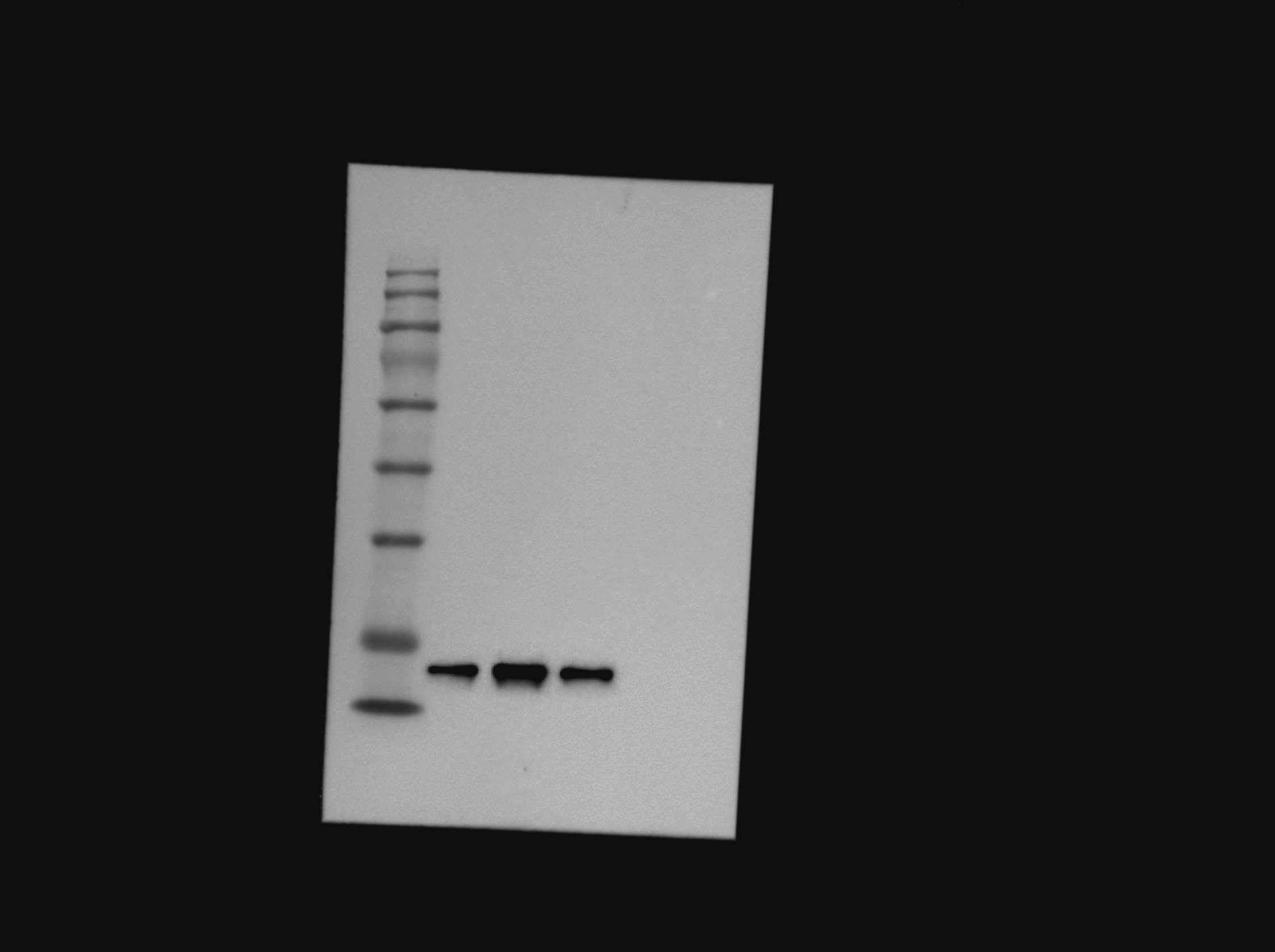


Figure 6I


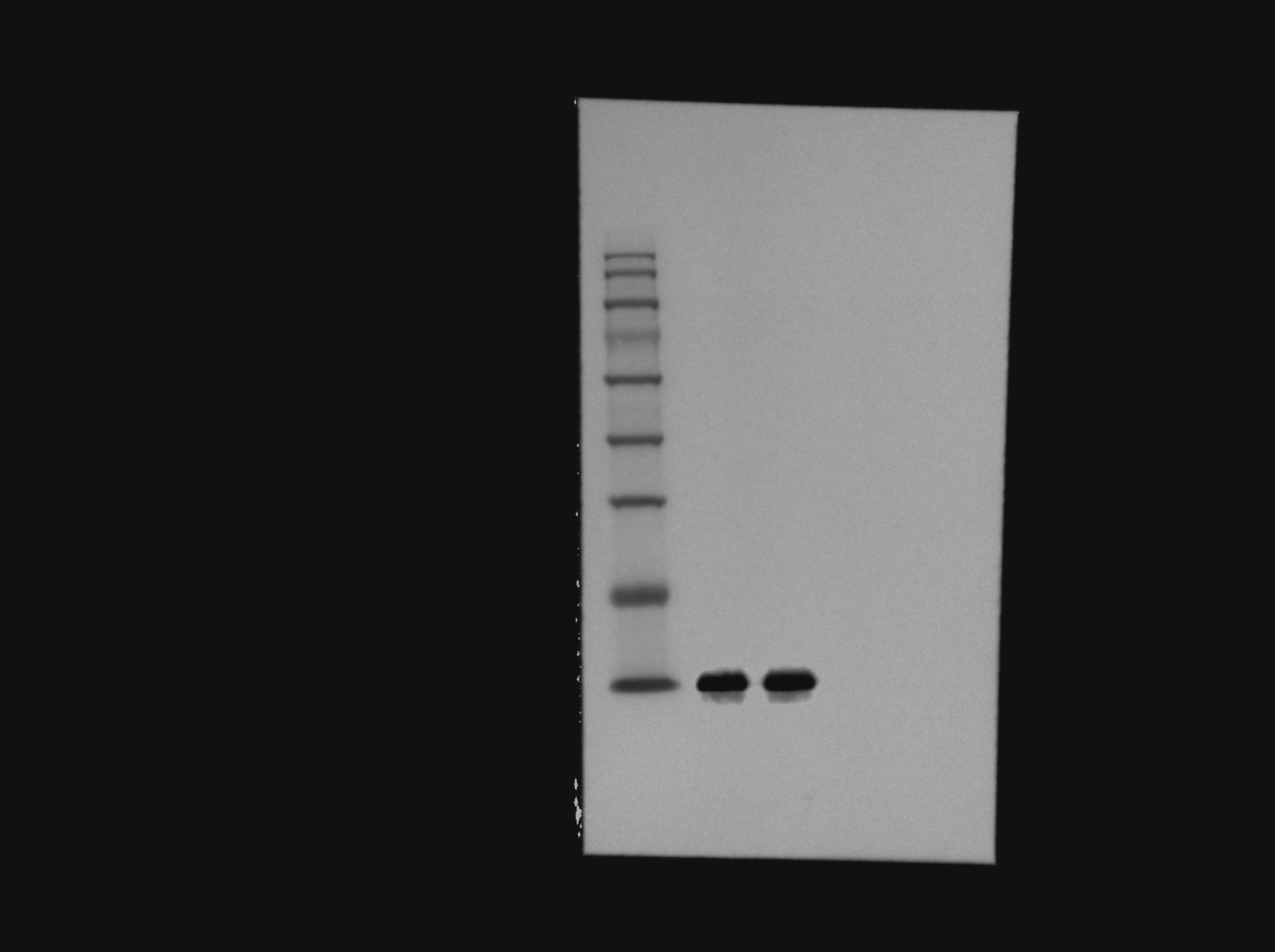

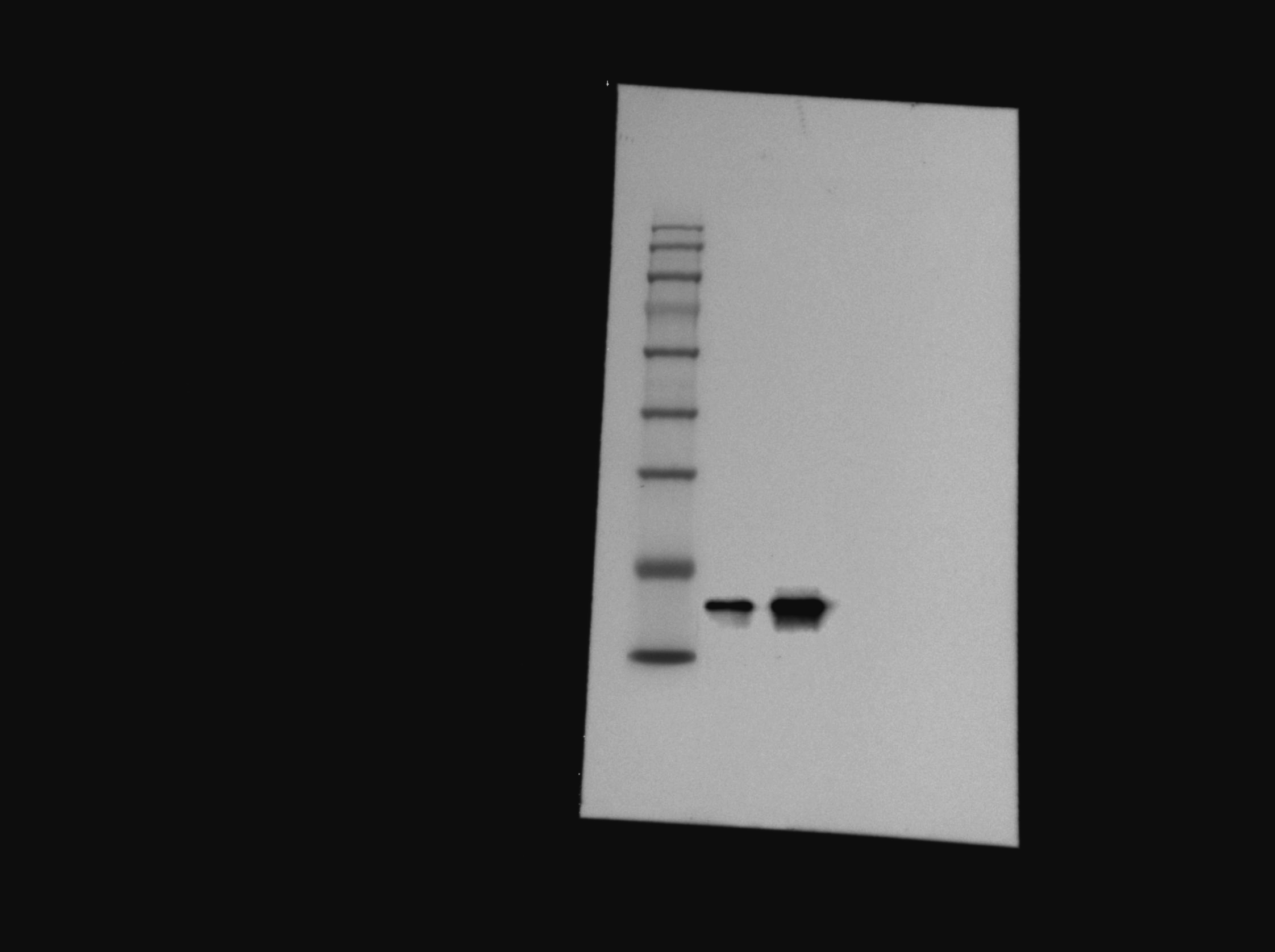

Supplement: Supplementary file 3 — Supplemental Material [file 41420_2022_1081_MOESM3_ESM.doc]
